# Parishin A-loaded mesoporous silica nanoparticles modulate macrophage polarization to attenuate tendinopathy

**DOI:** 10.1038/s41536-023-00289-0

**Published:** 2023-03-10

**Authors:** Lisha Zhu, Yu Wang, Shanshan Jin, Yuting Niu, Min Yu, Zixin Li, Liyuan Chen, Xiaolan Wu, Chengye Ding, Tianhao Wu, Xinmeng Shi, Yixin Zhang, Dan Luo, Yan Liu

**Affiliations:** 1 Laboratory of Biomimetic Nanomaterials, Department of Orthodontics, Peking University School and Hospital of Stomatology, Beijing, 100081 China; 2 Beijing Institute of Nanoenergy and Nanosystems, Chinese Academy of Sciences, Beijing, 101400 China; 3 National Center for Stomatology & National Clinical Research Center for Oral Diseases & National Engineering Laboratory for Digital and Material Technology of Stomatology & Beijing Key Laboratory of Digital Stomatology & Research Center of Engineering and Technology for Computerized Dentistry Ministry of Health & NMPA Key Laboratory for Dental Materials, Beijing, 100081 China; 4 Central Laboratory, Peking University School and Hospital of Stomatology, Beijing, 100081 China; 5 Department of Prosthodontics, Peking University School and Hospital of Stomatology, Beijing, 100081 China

**Keywords:** Drug delivery, Diseases

## Abstract

Macrophages are involved mainly in the balance between inflammation and tenogenesis during the healing process of tendinopathy. However, etiological therapeutic strategies to efficiently treat tendinopathy by modulating macrophage state are still lacking. In this study, we find that a small molecule compound Parishin-A (PA) isolated from Gastrodia elata could promote anti-inflammatory M2 macrophage polarization by inhibiting gene transcription and protein phosphorylation of signal transducers and activators of transcription 1. Local injection or sustained delivery of PA by mesoporous silica nanoparticles (MSNs) could almost recover the native tendon’s dense parallel-aligned collagen matrix in collagenase-induced tendinopathy by modulating macrophage-mediated immune microenvironment and preventing heterotopic ossification. Especially, MSNs decrease doses of PA, frequency of injection and yield preferable therapeutic effects. Mechanistically, intervention with PA could indirectly inhibit activation of mammalian target of rapamycin to repress chondrogenic and osteogenic differentiation of tendon stem/progenitor cells by influencing macrophage inflammatory cytokine secretion. Together, pharmacological intervention with a natural small-molecule compound to modulate macrophage status appears to be a promising strategy for tendinopathy treatment.

## Introduction

Tendons consist of parallel-aligned collagen fibers, which are responsible for transmitting tensile stresses from the muscle to the bone. Tendon diseases include acute injuries involving partial or complete tendon rupture, and chronic tendon disorders^1^, which frequently happen in athletes and groups occupied in high-strength musculoskeletal activities. Approximately 30 million tendon-related procedures are carried out annually in the world, producing a considerable socioeconomic burden^2^. Tendinopathy describes complex and diverse pathology of tendons that is commonly characterized by chronic tendon disorders, accompanied by disorganization of tendon matrix that could eventually result in tendon tears and ruptures^3^. It has been demonstrated that inflammation might drive tendon degeneration before tearing and lead to fibrovascular scarring during healing^4^. Notably, macrophages as versatile cells have been demonstrated to play a significant role in the inflammation reaction and tissue healing of tendinopathy by shifting to M1 phenotype or M2 phenotype^5^. Therefore, targeting blockade of macrophages in the early stage of tendinopathy is crucial to developing therapeutic strategies.

Meanwhile, macrophage infiltration and increased inflammatory cytokines have been suggested to disrupt tendon homeostasis, leading to pathological status and heterotopic ossification development^6^. Development of heterotopic ossification is another typical symptom of tendinopathy. A previous study has demonstrated that injured human tendons displayed higher levels of cartilage-related matrix genes and proteins^7^. Recently, studies have observed that untethered macrophage expansion and activation in tendinopathy trapped tendon stem/progenitor cells (TSPCs) into the inflammatory niche, which would coax TSPCs into abnormal chondrogenic and osteogenic differentiation^8^. For these reasons, the early therapeutic intervention of macrophage functions may be suitable methods for curbing inappropriate TSPC differentiation, ultimately protecting tendons from ectopic ossification.

Natural small molecule compounds, extracted from food ingredients, plants, or Chinese herbs, have various pharmacological activities, including anti-inflammatory, antioxidant, anti-parasite, and anti-virus effects^9–11^. Parishin A (PA) is an essential traditional Chinese medicine that belongs to a phenolic glucoside isolated from Gastrodia elata. It has been demonstrated that treatment with PA significantly ameliorated expressions of aging-related markers, such as growth differentiation factor 15, interleukin (IL)-6, and cyclin-dependent kinase inhibitor P16, indicating a potential modulatory effect of PA on inflammation^12^. Moreover, another parishin derivative, Parishin C, has been shown to efficiently reduce levels of inflammatory cytokines in vivo^13^. However, it is still unclear whether PA could directly regulate macrophage functions, thereby preventing the progression of tendinopathy.

In this study, we found that intervention with PA inhibited the expression of inflammatory factors and promoted M2 polarization of macrophages through Janus kinase-signal transducer and activator of transcription (JAK-STAT) signaling pathway. Using a collagenase type I-induced rat tendinopathy model, we found that local injection or sustained releasing of PA by mesoporous silica nanoparticles (MSNs) accelerated the healing of tendinopathy by inhibiting inflammation infiltration and promoting M2 macrophage polarization. Especially, MSNs decreased doses of PA, reduced injection frequency, and yielded preferable therapeutic effects. Moreover, delivery of PA also prevented the progression of heterotopic ossification by regulating macrophage inflammatory cytokine secretion, which was attributed to the inhibitory effect on the overactivation of mammalian target of rapamycin (mTOR) pathway in TSPCs. Our findings showed that pharmacological intervention via manipulating macrophage polarization ameliorated the inflammatory environment in tendinopathy and regulated TSPC behaviors to prevent heterotopic ossification. PA combined with a mesoporous silica delivery system appears to be a promising approach to prevent and treat other connective tissue diseases besides tendinopathy.

## Results

### PA promotes macrophage switch from pro-inflammatory M1 state to anti-inflammatory M2 state through JAK/STAT1 pathway

**Fig. 1:**
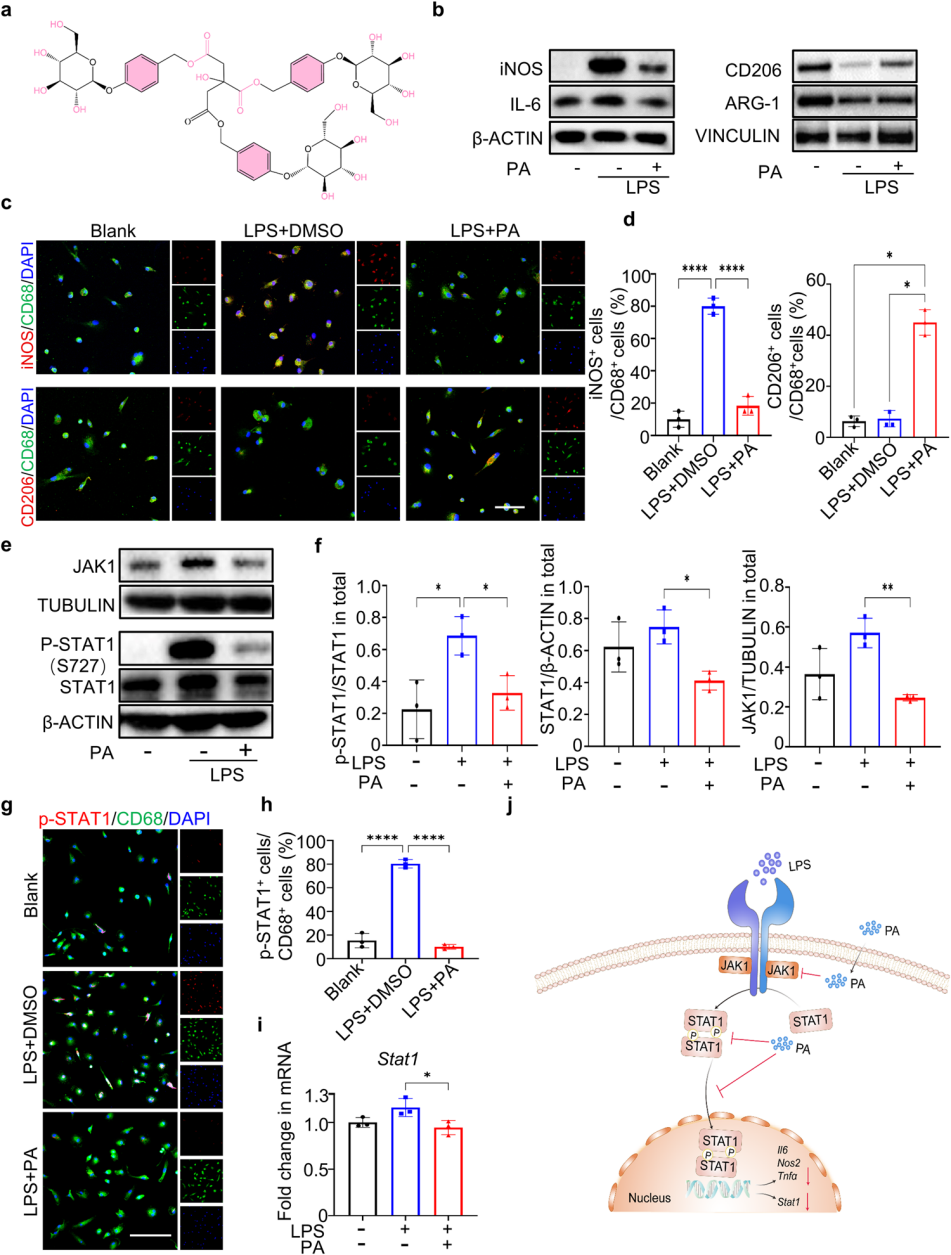
PA promotes M2 macrophage polarization through JAK/STAT1 pathway. **a** Molecular structure of the small-molecule compound PA. **b** Western blotting of iNOS, IL-6, CD206 and Arg-1 in DMSO- and PA-treated rat BMDMs under LPS stimulation. **c** Co-immunofluorescence staining for iNOS/CD68 and CD206/CD68 in unstimulated BMDMs and DMSO, PA-treated BMDMs under LPS stimulation. Scale bar, 50 μm. **d** Semi-quantification of **c**. **e** Western blotting of total JAK1, phosphorylation of STAT1 and total STAT1 in DMSO- and PA-treated BMDMs under LPS stimulation. **f** Semi-quantification of (**e**). **g** Immunofluorescence staining of p-STAT1 in DMSO- and PA-treated BMDMs under LPS stimulation. **h** Semi-quantification of (**g**). **i** RT-PCR of *Stat1* in expression in DMSO- and PA-treated BMDMs under LPS stimulation for 15 min. Scale bar, 50 μm. **j** Schematic illustration of the pharmacological mechanism of PA. Data are presented as mean ± SD. *p < 0.05, **p < 0.01 and ****p < 0.0001, *n* = 3.

Parishin A is constituted of three gastrodin molecules esterified with one terminal carboxyl group of citric acid^14^. The chemical characterization of PA was recorded by Fourier transform infrared spectroscopy (FTIR) (Fig. 1a and Supplementary Fig. 1a). The broad peaks at 3440 and 2925 cm^−1^ corresponded to the stretching vibrations of –OH and –CH, respectively. Characteristic multi-peaks at 1154, 1079, and 1024 cm^−1^ became stronger, representing the stretching vibration of C-O-C and C-O-H on the pyran ring. To screen for appropriate concentration and optimal anti-inflammation effects of PA, live/dead staining and macrophage polarization experiments were performed (Supplementary Fig. 1b–d). Treatment with 20 μM PA showed good biocompatibility to a cycling monocyte-derived macrophage, rat bone marrow-derived macrophages (BMDMs), and meanwhile exhibited optimal anti-inflammation effects, evidenced by decreased expression of inducible nitric oxide synthase (iNOS) and enhanced expression of arginase 1 (ARG-1). Therefore, we chose 20 μM PA in the following in vitro experiments. To explore the effect of PA on macrophage polarization, we stimulated BMDMs with lipopolysaccharide (LPS) to simulate an inflammatory environment. Western blotting results revealed that PA treatment efficiently inhibited the protein levels of inflammatory factors IL-6 and iNOS, and enhanced protein levels of anti-inflammatory factors CD206 and ARG-1 of LPS-stimulated BMDMs, compared to BMDMs treated with dimethyl sulphoxide (DMSO) (Fig. 1b, Supplementary Fig. 2). Further, we performed immunofluorescence staining of M1 and M2 macrophage markers to examine phenotypic alterations in macrophages under PA intervention. PA treatment efficiently reduced the ratio of CD68^+^iNOS^+^ M1 macrophages, as followed by an increase of CD68^+^CD206^+^ M2 macrophages. This finding indicates that PA treatment could impel BMDMs to shift from a M1 phenotype toward a M2 phenotype (Fig. 1c, d).

JAK-STAT family has been accepted as a classical pathway that influences all kinds of inflammation-related diseases by mediating macrophage functions^15,16^. More importantly, the inactivation of JAK-STAT pathway could efficiently inhibit inflammation infiltration in tendinopathy or senescence-induced inflammation secretion in aged TSPCs^17,18^. Hence, we hypothesized that PA modulated macrophage polarization through JAK/STAT1 pathway. Western blotting showed that LPS enhanced protein expression levels of JAK and phosphorylation of STAT1 (p-STAT1) in BMDMs, and PA treatment could remarkably reduce the JAK and p-STAT1 expression (Fig. 1e, f). Immunofluorescence also revealed that treatment with PA could efficiently suppress the nuclear expression of STAT1 (Fig. 1g, h). Noticeably, PA-treated BMDMs displayed reduced STAT1 protein levels compared to LPS-stimulated BMDMs. Therefore, we investigated the mRNA expression of STAT1 in all groups. Reverse transcription-polymerase chain reaction (RT-PCR) showed that treatment with PA evidently suppressed *Stat1* gene transcription (Fig. 1i), indicating that PA might modulate macrophage polarization through regulating the gene transcription of *Stat1* and phosphorylation levels of STAT1 protein.

Similar results were found in another cycling monocyte-derived macrophage, human THP-1-derived macrophage. PA treatment suppressed LPS-induced activation of pro-inflammatory factors at both mRNA and protein levels in human THP-1-derived macrophages (Supplementary Fig. 3a–c). Further, western blotting and immunofluorescence revealed that PA also inhibited JAK-STAT signaling with reduced phosphorylation of *STAT1* (Supplementary Fig. 3d, e). Results from human cells further excluded casual consequence that only happened in cells from animal species. Taken together, PA efficiently inhibited M1 macrophage activation and promoted M2 macrophage polarization via JAK/STAT1 signaling pathway (Fig. 1j).

### PA alleviates collagenase-induced tendinopathy by modulating macrophage-mediated immune microenvironment

**Fig. 2:**
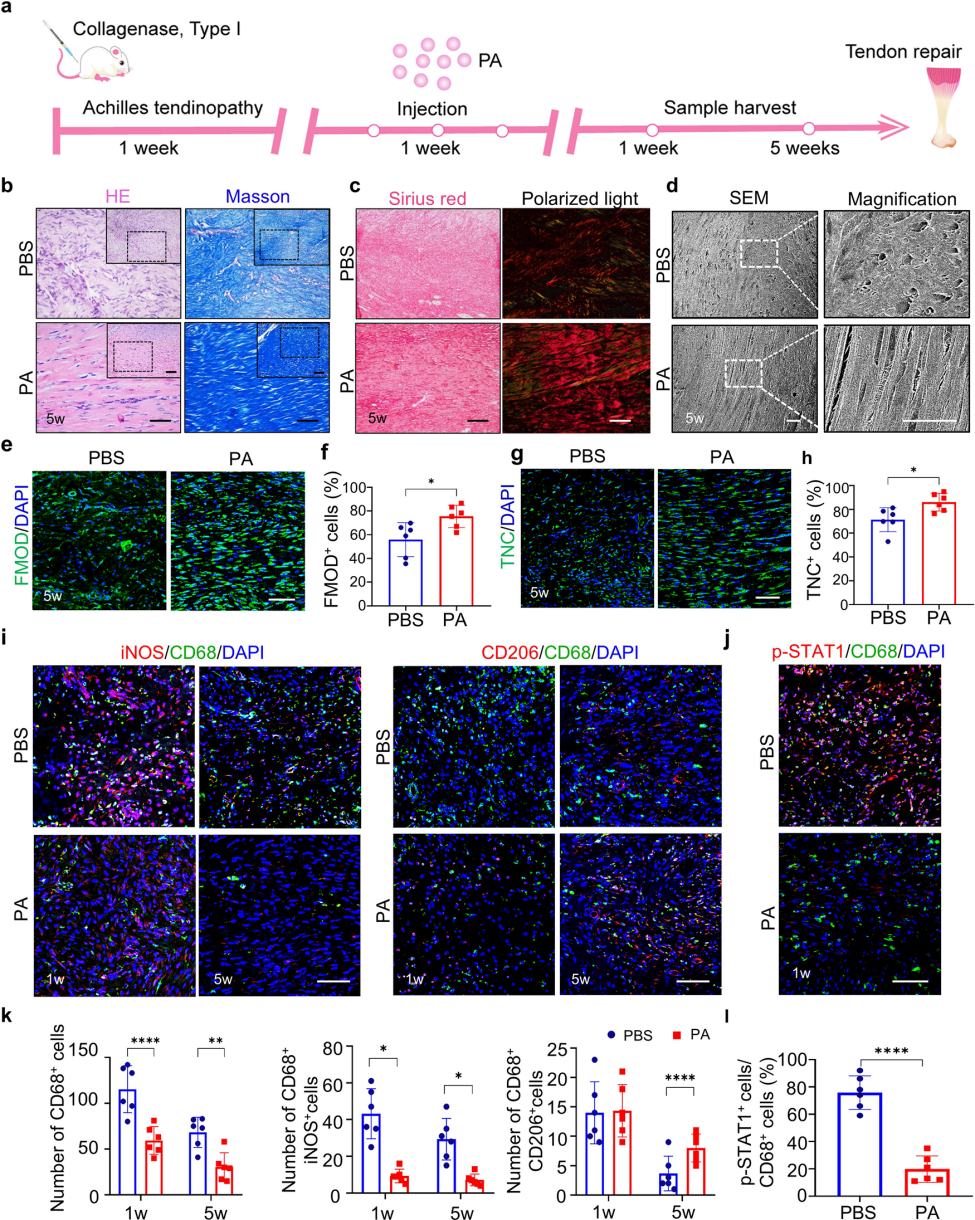
PA alleviates collagenase-induced tendinopathy by inhibiting inflammatory response. **a** Schematic illustration of the experimental animal procedure. **b** HE and Masson’s trichrome staining of neo-tendons from PBS and PA groups at 5 weeks. Scale bar, 100 μm. **c** Representative Sirius red staining and polarized light images showing the collagen type in neo-tendons from PBS and PA groups at 5 weeks. (Green, type III collagen; red, type I collagen). Scale bar, 100 μm. **d** SEM of collagen pattern of newly-formed tendon collagen fibrils in each group at 5 weeks. Scale bar, 40 μm. **e** Immunofluorescence staining of tenogenic marker FMOD in neo-tendons from PBS and PA groups at 5 weeks. Scale bar, 100 μm. **f** Semi-quantification of **e**. **g** Immunofluorescence staining of tenogenic marker TNC in neo-tendons from PBS and PA groups at 5 weeks. Scale bar, 100 μm. **h** Semi-quantification of (**g**). **i** Co-immunofluorescence staining of iNOS and CD68, and CD206 and CD68 in neo-tendons from PBS and PA-treated groups at 1 week and 5 weeks. Scale bar, 100 μm. **j** Immunofluorescence staining of p-STAT1 and CD68 in 1w neo-tendons from PBS and PA-treated groups at 1 week. Scale bar, 100 μm. **k** Semi-quantification of total CD68^+^, iNOS^+^CD68^+^ and CD206^+^CD68^+^ cells in (**i**). **l** Semi-quantification of (**j**). Data are presented as mean ± SD. *p < 0.05, **p < 0.01 and ****p < 0.0001, *n* = 6.

Tendinopathy is characterized by inflammation infiltration and heterotopic ossification. Persistent inflammation aggravates the progression of tendinopathy^19^. It has been widely known that macrophages play an essential role in the development of tendinopathy. Timely intervention to transform macrophages into an anti-inflammation condition more swiftly is a reliable modulative strategy to alleviate tendinopathy symptoms^4^. The above in vitro results demonstrated that treatment with PA inhibited the expression of inflammatory factors and promoted macrophage switch into the M2 subtype more swiftly. Thus, we further investigated whether PA could modulate macrophage polarization to reverse inflammation by using a collagenase-induced rat tendinopathy model (Fig. 2a). Injection of type I collagenase resulted in conspicuous swelling in the hindlimb, which was lessened by the following injection with PA solution three times a week (Supplementary Fig. 4a). Haematoxylin-eosin (HE) and Masson’s trichome stainings revealed that delivery of PA mitigated the development of tendon degeneration after 7 days of injection, including alleviative disorganization of tendon fibers and reduced infiltration of inflammatory cells (Supplementary Fig. 4b). Next, we examined the progression of tendinopathy in the phosphate buffer solution (PBS) and PA groups following 5 weeks of injection, which reached adaptive remodeling of the tendon matrix. HE and Masson’s trichome stainings showed that tendons from the PA group presented more compact and orderly alignment of collagen deposition than those from the PBS control group (Fig. 2b). Sirius red staining showed that tendons from the PA group displayed a bright red color and continuously oriented morphology, whereas the tissues from the PBS group exhibited relatively irregular arrangement (Fig. 2c). The aligned collagen pattern could be a predictor of good mechanical properties and healing of tendons. Scanning electron microscope (SEM) also revealed that delivery of PA yielded extracellular matrix with greater density and more parallel arrangement at 5 weeks (Fig. 2d). Furthermore, histological slides were interpreted using the semi-quantitative grading scale of Movin score^20^, assessing various aspects of tendon tissues. Compared with the control group, tendon tissues in the PA group showed a much lower Movin score, indicating a significant improvement in fiber structure and arrangement, increased vascularity, decreased collagen stainability and the glycosaminoglycan content (Supplementary Table 1). Moreover, immunofluorescence showed more robust expression of tenogenic markers fibromodulin (FMOD) and Tenascin-C (TNC) in tendons from the PA group compared to those from the PBS group (Fig. 2e–h).

To investigate whether better tendon repair is attributed to PA regulation on macrophage polarization in vivo, we examined the inflammation infiltration condition in both groups. Immunofluorescence showed that delivery of PA significantly boosted the recession of CD68 positive macrophages in the PA group (Fig. 2i, k). More importantly, there were decreased amounts of CD68^+^iNOS^+^ macrophages and increased amounts of CD68^+^CD206^+^ macrophages in the PA group compared to the PBS group. This indicates that intervention with PA promotes macrophage polarization from M1 to M2 subtypes more swiftly. Mechanistically, PA promoted tendon repair in vivo by boosting the macrophage switch from the M1 to M2 subtype via the STAT1 pathway, evidenced by reduced CD68^+^STAT1^+^ macrophages in the PA group at 1 week of operation (Fig. 2j, l).

### PA prevents tendon heterotopic ossification by inhibiting TSPC aberrant differentiation and attenuating pro-inflammatory cytokine secretion in macrophages

**Fig. 3:**
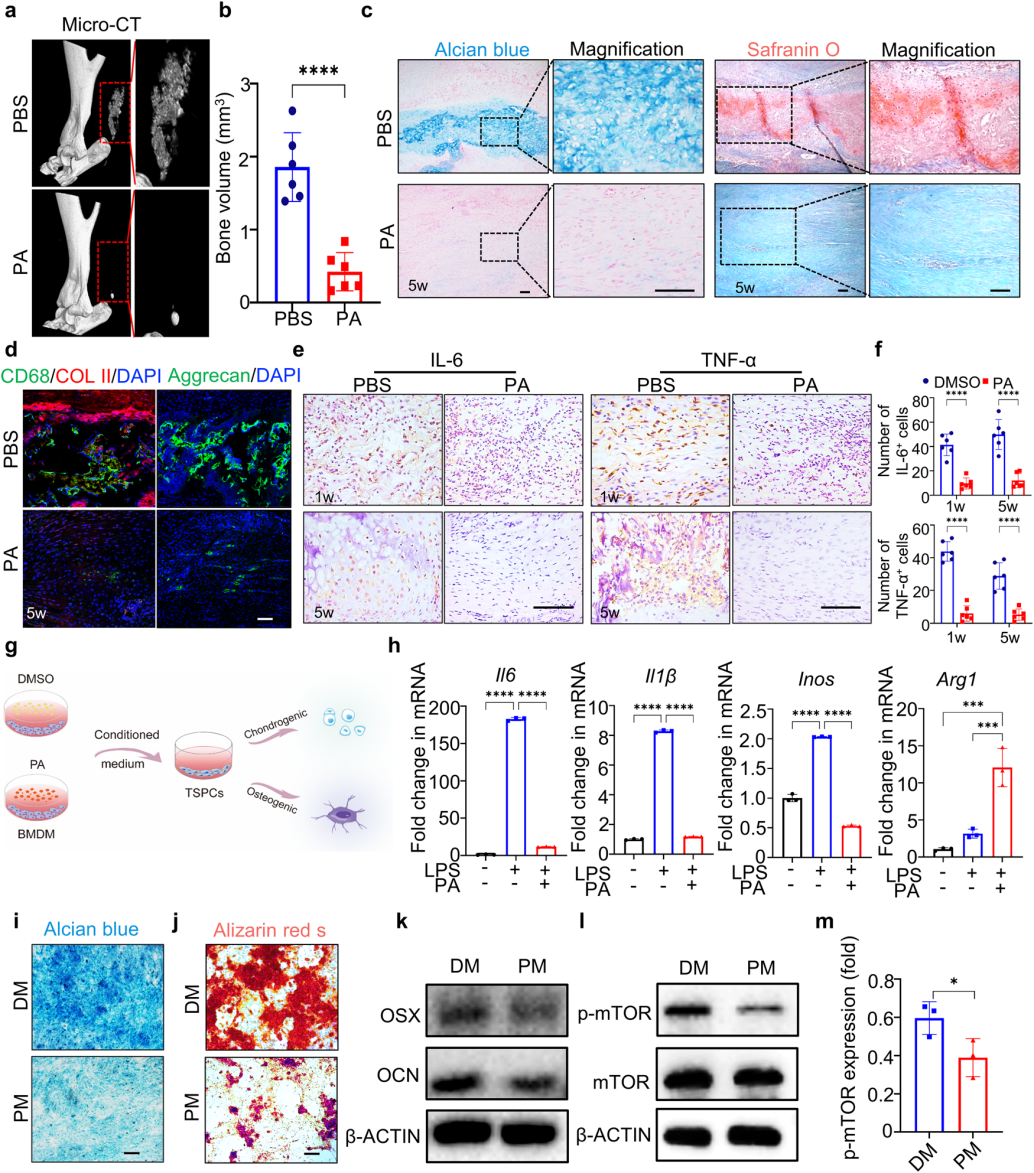
PA prevents tendon heterotopic ossification through inhibiting aberrant differentiation of TSPCs by attenuating pro-inflammatory cytokine secretion in macrophages. **a** Micro-CT images of repaired tendons from PBS and PA groups at 5 weeks. **b** Semi-quantification of (**a**). Data are presented as mean ± SD. ****p < 0.0001, *n* = 6. **c** Alcian blue staining and Safranin O staining images of repaired tendons from PBS and PA groups at 5 weeks. Scale bar, 100 μm. **d** Immunofluorescence staining of chondrocyte markers COL II and Aggrecan in neo-tendons from PBS and PA groups at 5 weeks. Scale bar, 100 μm. **e** Immunohistochemical staining of IL-6 and TNF-α in neotendons from PBS and PA groups at 1 and 5 weeks. Scale bar, 100 μm. **f** Semi-quantification of **e**. Data are presented as mean ± SD. ****p < 0.0001, *n* = 6. **g** Schematic illustration of the process for inducing TSPCs chondrogenic and osteogenic differentiation. **h** RT-PCR of *Il6*, *Il1β*, *Inos*, and *Arg1* expression in DMSO- and PA-treated BMDMs under LPS stimulation. Data are presented as mean ± SD. ***p < 0.001 and ****p < 0.0001, *n* = 3. **i** Alcian blue staining of TSPCs under chondrogenic differentiation for 14 days. Scale bar, 100 μm. **j** ARS staining of TSPCs under osteogenic differentiation for 21 days. Scale bar, 100 μm. **k** Western blotting of OSX and OCN in TSPCs. **l** Western blotting of p-mTOR in TSPCs at 7 days. **m** Semi-quantification of **l**. Data are presented as mean ± SD. *p < 0.05, *n* = 3. DM means conditioned medium from DMSO-treated BMDMs under LPS stimulation; PM means conditioned medium from PA-treated BMDMs under LPS stimulation.

Accumulation of cartilage-like or bone-like matrix consisting of ground substances is a hallmark of tendinopathy^21^. Micro-CT showed that tendons from the PBS group exhibited high mineral density with a bone volume of ~1.86 mm^3^, whereas treatment with PA significantly reduced mineral density with a bone volume of ~0.42 mm^3^ (Fig. 3a, b). Furthermore, HE, Masson’s trichome, Alcian blue, and Safranin O stainings revealed typical endochondral ossification in the PBS group, whereas PA treatment prevented tendon heterotopic ossification (Fig. 3c, Supplementary Fig. 5a). Immunofluorescence showed lower expression of chondrocyte markers, collagen II and aggrecan were found in the PA group compared to the PBS group (Fig. 3d). Interestingly, large amounts of CD68-positive macrophages were located around the cartilage-like tissues, indicating that inflammation from macrophages was a crucial cause of abnormal cartilage deposition. Moreover, immunohistochemical staining also showed that intervention with PA reduced the expression of inflammatory factors IL-6 and tumor necrosis factor α (TNF-α) at both 1 week and 5 weeks (Fig. 3e, f). Here, we showed that administration of PA primarily reduced heterotopic ossification besides the inhibition of inflammation.

It has been accepted that TSPCs, when situated in inflammatory niche of tendinopathy, would present a strong tendency to differentiate into chondroid and osteoid tissues^7^. Our in vivo experiments showed that PA inhibited the secretion of inflammation cytokines, which may influence the living environment of TSPCs in vivo and result in their aberrant differentiation. Surely, the specific cytokines produced by PA-stimulated macrophages in vivo may need further study. In order to explore whether PA affected TSPC differentiation by directly influencing macrophage inflammatory cytokine secretion, we collected the culture supernatant of LPS-stimulated BMDMs under PA or DMSO treatment. Then, we supplemented these two types of the conditioned medium into TSPC culture respectively and compared their effects on chondrogenic and osteogenic differentiation of TSPCs (Fig. 3g). RT-PCR results revealed that reduced mRNA expression levels of inflammatory factors *Il6*, *Il1β*, and Inos in the conditioned medium from PA-treated macrophages, but increased expression levels of anti-inflammatory factors *Arg1* (Fig. 3h). Alcian blue staining showed significantly decreased stained area of chondroid tissues in PA-conditioned medium compared to the DMSO group (Fig. 3i). As for osteogenic differentiation of TSPCs, the conditioned medium from PA-treated macrophages inhibited the formation of calcium nodules and downregulated osteogenesis-related protein levels Osterix (OSX) and osteocalcin (OCN) (Fig. 3j, k, Supplementary Fig. 5b). Aberrant activation of the mTOR signaling pathway has been demonstrated to result in biased differentiation of TSPCs and cause abnormal heterotopic ossification in tendinopathy^7,22^. Western blotting showed that the conditioned medium from PA-treated macrophages markedly inhibited the phosphorylation levels of mTOR in TSPCs (Fig. 3l, m). Overall, these findings indicate that PA efficiently prevented heterotopic ossification *via* indirectly curbing biased differentiation of TSPCs that was influenced by pro-inflammatory cytokine secretion in macrophages.

### Sustained release of PA based on a MSN nano-carrier system

It has been demonstrated that MSNs as drug carriers have the potential to augment local drug concentrations in inflamed tissues, improve drug efficacy, decrease dosing frequency, and reduce side effects^23,24^. Therefore, to improve the PA dissolution rate and further heighten the possibility of clinical translation, we utilized the MSNs, approved by the US Food and Drug Administration, as drug carriers to provide sustained release of insoluble PA at sites of tendon damage.

**Fig. 4:**
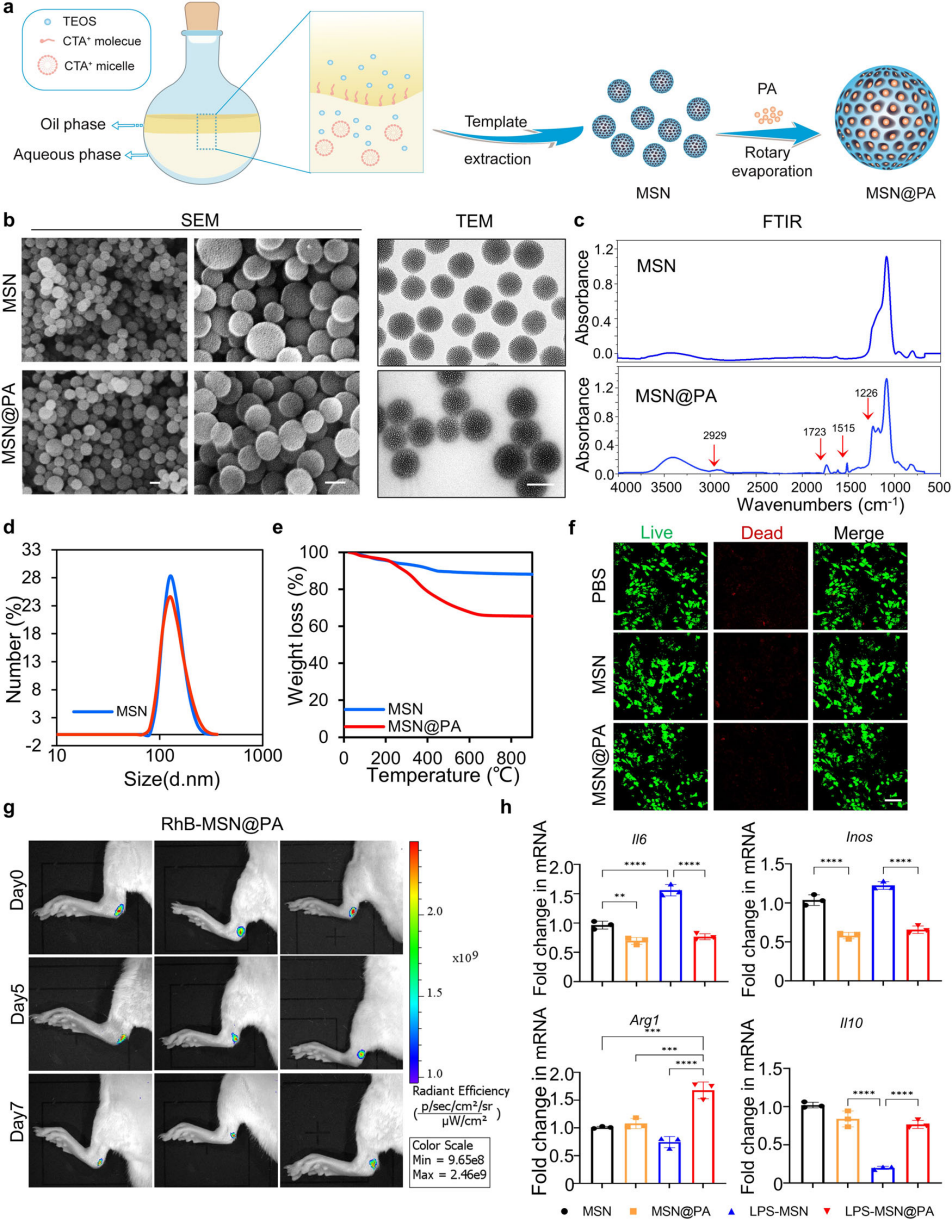
Characterization of MSN@PA. **a** Scheme of synthesis process for MSN@PA. **b** TEM and SEM images of MSNs and MSN@PA. Scale bar, 100 nm. **c** FTIR spectra of MSNs and MSN@PA. **d** DLS analysis of MSNs and MSN@PA. **e** TGA of MSNs and MSN@PA. **f** Live/dead staining of BMDMs treated by MSN@PA for 48 h. Scale bar, 100 μm. **g** In vivo imaging of the rat Achilles tendon after nanoparticles injection at 0 day, 5 days, 7 days. **h** RT-PCR of *Il6*, *Inos*, *Arg1*, and *Il10* expression in unstimulated BMDMs and MSNs, MSN@PA -treated BMDMs under LPS stimulation for 12 h. Data are presented as mean ± SD. **p < 0.01, ***p < 0.001 and ****p < 0.0001, *n* = 3.

MSNs were prepared via a one-pot biphase stratification approach to achieve generational and center-radial mesopore channels^25,26^. After removing the residual reactants, PA-loaded nanoparticles (MSN@PA) were ultimately achieved by solvent evaporation technique (Fig. 4a). The obtained MSN@PA were spherically shaped with mesoporous/macroporous structure and maintained typical structures of MSNs during the drug loading process, as evidenced by SEM and transmission electron microscopy (TEM) images (Fig. 4b). Compared to MSNs (~166 nm), the hydrodynamic diameter of MSN@PA changes a little, as observed by dynamic light scattering (DLS) and TEM, indicating that PA was loaded at the inner core of MSNs (Fig. 4b, d). The FTIR spectra showed that MSN@PA contained the characteristic peaks of MSNs and PA, indicating that PA was successfully loaded into the nanomotors (Fig. 4c). Furthermore, thermal gravimetric (TG) measurement was used to determine the mass ratio of loaded PA on MSNs. Results revealed that the mass ratio of loaded PA on MSNs was about 23% (Fig. 4e).

Firstly, we investigated the biological safety of MSN@PA. Live/dead staining indicated that MSNs exhibited no evident cytotoxicity to rat BMDMs with concentrations ranging from 0 to 50 μg/mL (Supplementary Fig. 6b). Moreover, we found that 50 μg/mL MSN@PA also presented no cytotoxicity on rat TSPCs (Fig. 4f). Therefore, we chose 50 μg/mL as loaded dose of PA on MSNs to be applied in follow-up experiments. Since sustained releasing of drugs is beneficial to enhance the therapeutic effect in vivo, we next examined the accumulated drug-releasing pattern of MSN@PA. Results showed that MSN@PA possessed a sustained release rate and could last for at least 11 days (Supplementary Fig. 6a).

Further, we investigated the biodistribution of free MSN@PA in rat Achilles tendon by in vivo imaging (Fig. 4g). Notably, the strong fluorescence of Rhodamine B (RhB, red fluorescence)-labeled free MSN@PA was observed in tendons at 7 days. The retention of nanoparticles was good for effectively PA accumulation in local tendon tissue, avoiding drug leakage. While the drug-releasing pattern might be attributed to the large size and mesoporous/macroporous structure^24^. Based on the regulatory role of PA on macrophage polarization, the potential of MSN@PA as a modulator of macrophage polarization in vitro was systematically evaluated. As shown in Fig. 4h, MSN@PA effectively suppressed inflammation, in part, by activating an M2 macrophage phenotype. Together, these results confirmed that MSN@PA modulated macrophage functions in vitro.

### MSN@PA effectively promotes tendon repair by targeting macrophages

**Fig. 5:**
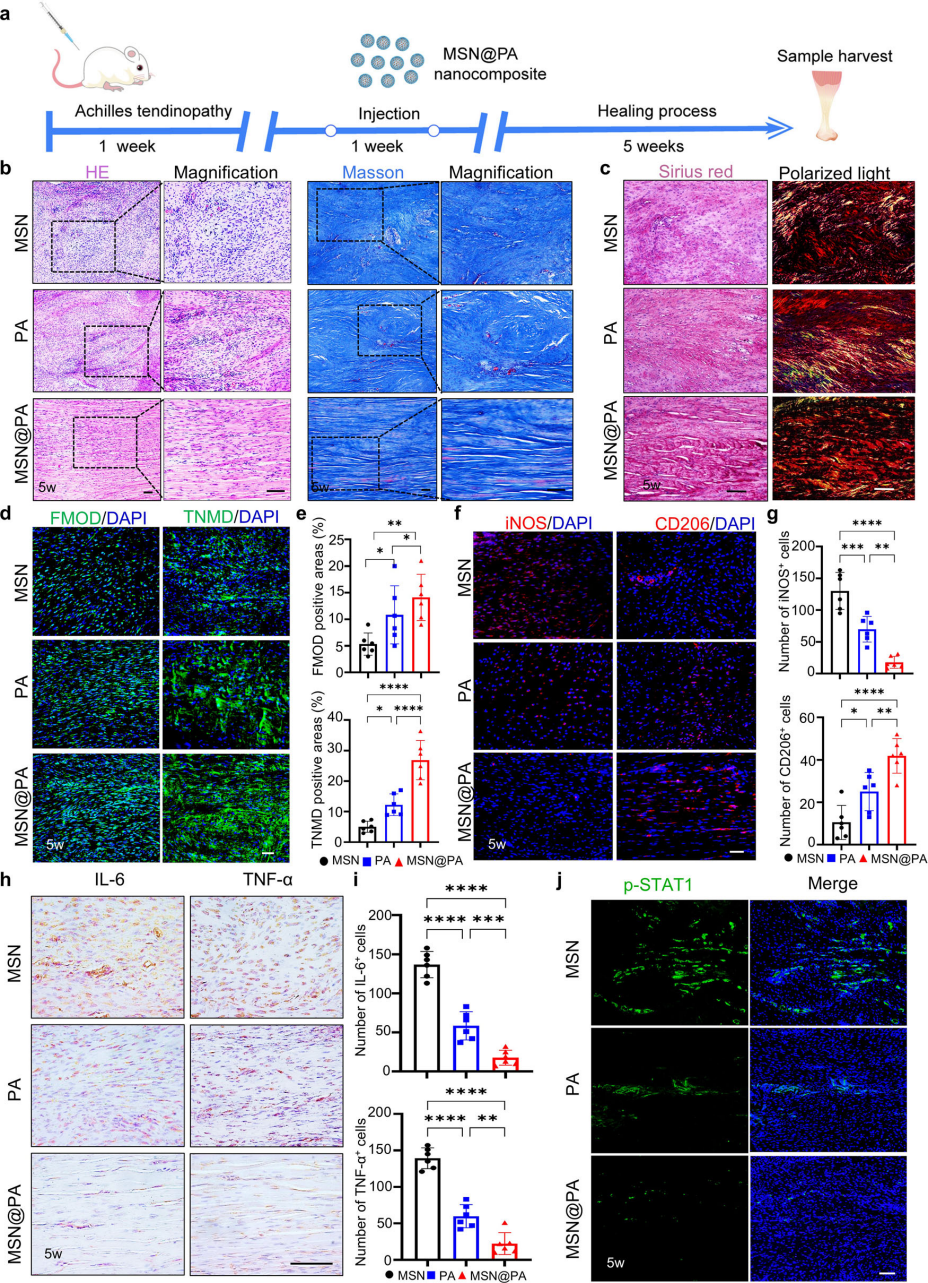
Local injection of MSN@PA with reduced frequency promotes recovery of tendinopathy by boosting macrophage polarization. **a** Scheme of local delivery design. **b** HE and Masson’s trichrome stainings of neo-tendons from MSNs, PA, and MSN@PA groups at 5 weeks. Scale bar, 100 μm. **c** Representative Sirius red staining and polarized light images of repaired tendons from the PBS and PA groups at 5 weeks. Scale bar, 100 μm. **d** Immunofluorescence staining of FMOD and TNMD in repaired tendons from the PBS and PA groups at 5 weeks. Scale bar, 100 μm. **e** Semi-quantification of **d**. **f** Immunofluorescence staining of iNOS and CD206 in repaired tendons from the PBS and PA groups at 5 weeks. Scale bar, 100 μm. **g** Semi-quantification of **f**. **h** Immunohistochemical staining of IL-6 and TNF-α in repaired tendons from PBS and PA groups at 5 weeks. Scale bar, 100 μm. **i** Semi-quantification of **h**. **j** Immunofluorescence staining of p-STAT1 in repaired tendons from the PBS and PA groups at 5 weeks. Scale bar, 100 μm. Data are presented as mean ± SD. *p < 0.05, **p < 0.01, ***p < 0.001 and ****p < 0.0001, *n* = 6.

To examine the therapeutic effects on tendinopathy, MSN@PA was administrated in situ after the injection of collagenase type I. Notably, given the sustained-releasing capacity of MSN@PA in vitro, we reduced the injection frequency from three times a week to twice a week (Fig. 5a). After 5 weeks of operation, animals were sacrificed for further histological evaluations. HE and Masson’s trichome stainings indicated that tendons from the MSN group displayed severe collagen disorders and apparent inflammatory infiltration. In contrast, the MSN@PA group possessed aligner and thicker collagen fiber and decreased inflammatory infiltration (Fig. 5b, Supplementary Table. 2). Meanwhile, HE staining suggested that MSN@PA did not cause histological toxicity to the principal organs of rats (Supplementary Fig. 7). Noticeably, compared to tendons that received an injection of PA three times a week, the MSN@PA group displayed better recovery in histological structure. Sirius red staining and polarized light imaging revealed that tendons from the MSN@PA group have more ordered fiber alignment and mature collagen composition compared to the MSNs and PA groups (Fig. 5c). Furthermore, immunofluorescence showed that tendons from the MSN@PA group displayed higher expression of tenogenic markers FMOD and Tenomodulin (TNMD) in repaired tendons (Fig. 5d, e). Above examinations demonstrated that injection of MSN@PA twice a week could achieve a faster and better therapeutic effect, which may reduce the side effect due to multiple invasive injections.

Tendinopathy is often accompanied by persistent inflammation insult in tendon injury. To investigate whether the preferable effects of MSN@PA are attributed to its modulative effect on macrophages in vivo, we further examined the typical M1 and M2 macrophage markers. Immunofluorescence and immunohistochemistry showed decreased expression of inflammation factors, such as iNOS, IL-6, and TNF-α, in the MSN@PA group, which was coupled with a reduced number of p-STAT1 positive cells (Fig. 5h–j). Interestingly, compared to the MSNs and PA group, much more CD206 positive cells were identified in repaired tendons from the MSN@PA group (Fig. 5f, g). Therefore, MSN@PA performed its macrophage-modulative function better in vivo to promote recovery of tendinopathy compared to a simple injection of PA.

**Fig. 6:**
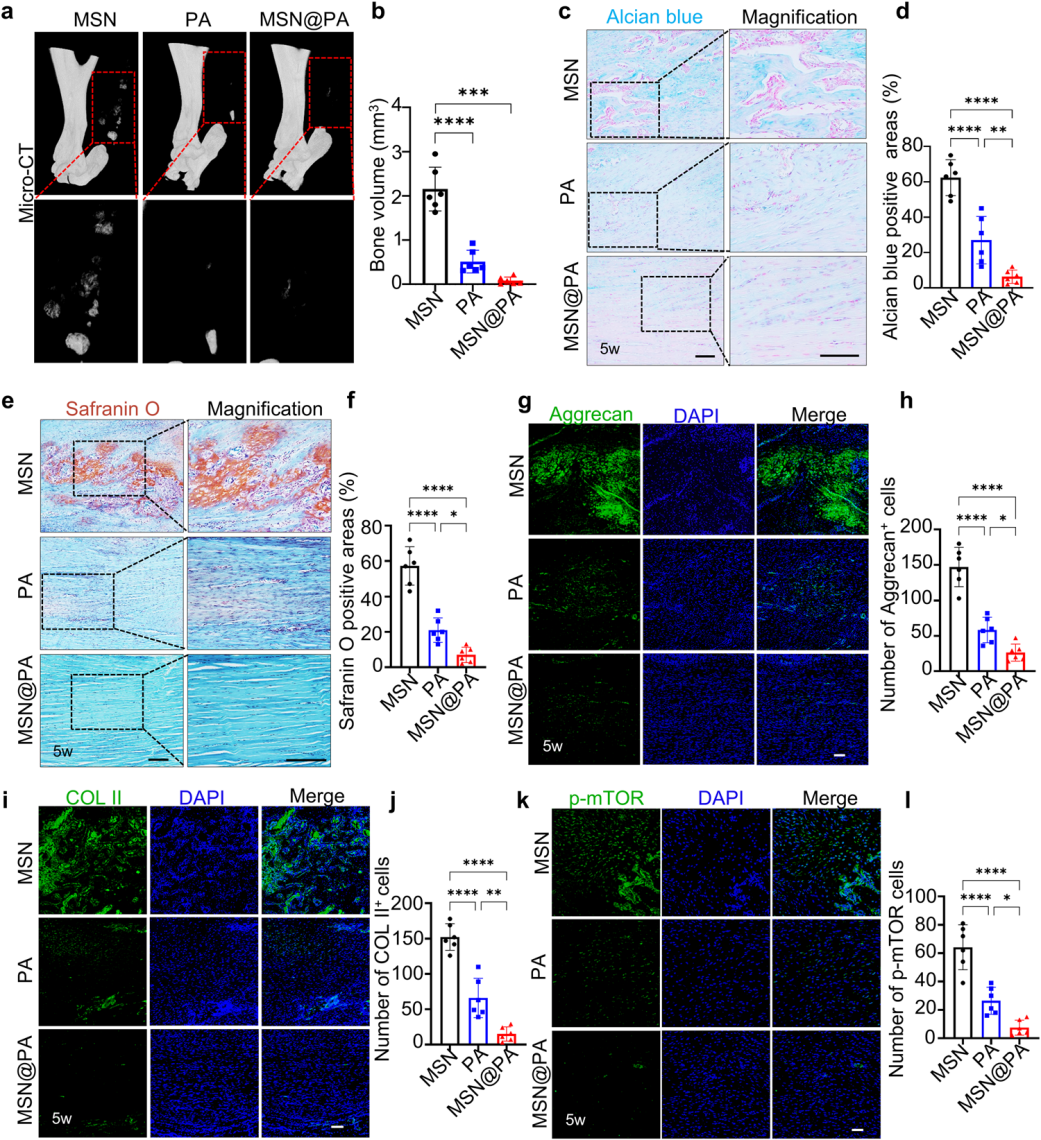
MSN@PA better prevents heterotopic ossification by inhibiting activation of mTOR. **a** Micro-CT scans of repaired tendons from the PBS and PA groups at 5 weeks. **b** Semi-quantification of high-intensity zones in **a**. **c** Alcian blue staining of repaired tendons from the PBS and PA groups at 5 weeks. Scale bar, 100 μm. **d** Semi-quantification of **c**. **e** Safranin O staining of repaired tendons from the PBS and PA groups at 5 weeks. Scale bar, 100 μm. **f** Semi-quantification of **e**. **g** Immunofluorescence staining of chondrocyte marker Aggrecan in repaired tendons from the PBS and PA groups at 5 weeks. Scale bar, 100 μm. **h** Semi-quantification of **g**. **i** Immunofluorescence staining of chondrocyte marker COL II in repaired tendons from the PBS and PA groups at 5 weeks. Scale bar, 100 μm. **j** Semi-quantification of **i**. **k** Immunofluorescence staining of p-mTOR repaired tendons from the PBS and PA groups at 5 weeks. Scale bar, 100 μm. **l** Semi-quantification of (**k**). Data are presented as mean ± SD. *p < 0.05, **p < 0.01, ***p < 0.001 and ****p < 0.0001, *n* = 6.

**Fig. 7:**
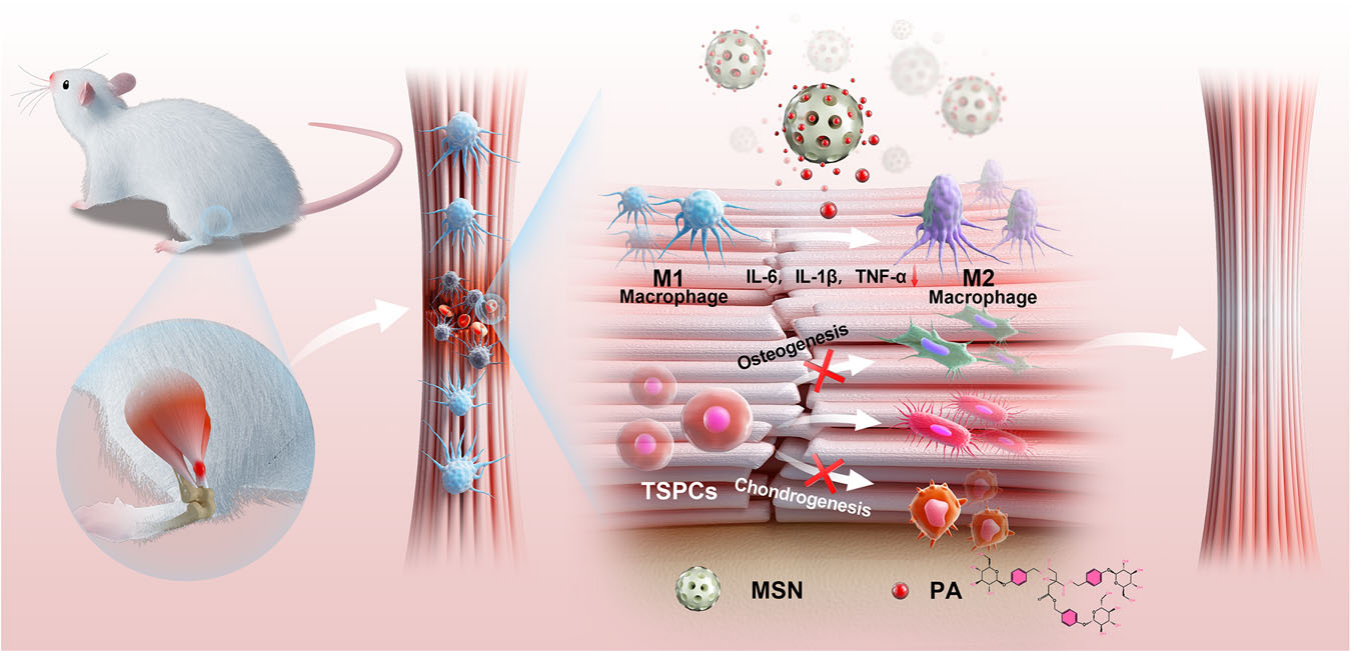
Schematic illustration of MSN@PA regulating macrophages to promote tendon repair. Sustained delivery of PA by mesoporous silica nanoparticles (MSNs) could almost recover the native tendon’s dense parallel-aligned collagen matrix in collagenase-induced tendinopathy by modulating macrophage-mediated immune microenvironment and preventing heterotopic ossification.

Next, we explored whether MSN@PA could prevent the progression of heterotopic ossification. Micro-CT indicated that MSN@PA significantly decreased the occurrence of heterotopic ossification in the injured tendons (Fig. 6a, b). Alcian blue and Safranin O stainings showed that injection of MSN@PA almost completely inhibited endochondral ossification (Fig. 6c–f). Results of Movin score showed that PA changed the pathologic progress, and improved the collagen parallel arrangement, the increase in vascularity and the decreased collagen stainability (Supplementary Table 2). Since chondroid tissues were not apparent in Micro-CT images, we further performed immunofluorescence to examine whether MSN@PA could also reduce chondroid tissues. Results showed that the MSN group still retained large areas of Aggrecan and COL II positive areas, whereas MSN@PA almost eliminated chondroid tissues compared to tendons from PA groups (Fig. 6g–i). Furthermore, we also analyzed the expression of p-mTOR in all groups and found that MSN@PA efficiently inhibited the activation of mTOR pathway (Fig. 6k, l). In general, MSN@PA effectively promoted tendon repair and prevented abnormal heterotopic ossification caused by an inflammatory microenvironment. Compared with direct injection, this PA delivery system exhibited the outstanding characteristic of sustained release (Fig. 7). The sustained release property greatly reduced the required drug injection frequency and improved the treatment’s effectiveness.

## Discussion

Tendinopathy, a type of tendon disorder, is characterized by chronic inflammation infiltration and heterotopic ossification. In this study, we illustrated that a small molecule compound Parishin A, a phenolic glucoside isolated from Chinese medicine Gastrodia elata, efficiently suppressed the conversion of monocytes and macrophages into a pro-inflammatory M1-like phenotype and promoted the transition into a pro-reparative M2-like phenotype through JAK/STAT1 pathway. By using a rat collagenase-induced tendinopathy model, we demonstrated that administration of PA efficiently promoted tendon repair and prevented the progression of heterotopic ossification. Furthermore, we developed a drug delivery system, MSN@PA composite that realized the drug’s therapeutic effect with sustained release feature and reduced in vivo injection frequency (Fig. 7). This drug delivery system was more effective in alleviating inflammation and heterotopic ossification, thereby permitting more efficient tendon repair in collagenase-induced tendinopathy.

Despite efforts to develop suitable therapy for tendinopathy, structural repair of tendons is still clinically challenging. Traditional treatments, including physical therapies^27^, non-steroidal anti-inflammatory drugs^28,29^, and surgery^30^ have been used for tendinopathy. While these treatments may substantially reduce some tendinopathy symptoms, etiological therapeutic strategies to address the underlying pathology in tendinopathy are still lacking. As increasing advancement in tendon biology, researchers have recognized that inflammation plays a vital role in the pathogenesis of tendinopathy^1,4^. Recently, small molecule compounds have been gradually applied in the treatment of various tissue repair and regeneration, and possess distinct advantages including convenience to use, no immune-rejection, and fine-tunable biological effects^10,11^. Increasing researches focus on developing small molecule drugs that can modulate intracellular signal pathways to specifically refine inflammation environment to promote tendon repair^31,32^. As is well known, macrophages increased markedly in tendon injury and exerted important functions in the development and progression of tendinopathy^33^. We therefore tried to regulate macrophage functions by a small molecule compound PA, to prevent the progression of tendinopathy. We found that PA promoted anti-inflammatory M2 macrophage polarization by inhibiting gene transcription and protein phosphorylation of STAT1. The JAK/STAT signaling pathway is regarded as one of the central communication nodes in the cell function^34^. It is implicated in the pathogenesis of inflammatory and autoimmune diseases including rheumatoid arthritis, psoriasis, and inflammatory bowel disease^35^. Consistently, we showed the suppressive effect of PA on gene transcription of the JAK and phosphorylation of STAT1 at S727. The negatively regulating mechanism of PA on transduction of the JAK/STAT signaling needs further exploration. Additionally, the current era of single-cell analysis is rapidly changing the understanding of macrophages. Some resident macrophages are not only derived from embryonic precursors, but also can be differentiated from circulating monocytes^36^. Under pathological conditions, the proportion of monocyte-related macrophages increases, and these macrophages play an important regulatory role in tendon repair^37^. Therefore, we chose cycling monocyte-derived macrophages BMDMs and THP-1 cells as in vitro experimental models, and consistent results of macrophage polarization by PA were found. Combined with the effect of PA on CD68^+^ macrophages in vivo, we proved that PA promoted M2 macrophage polarization and thereby attenuated collagenase-induced tendinopathy. Of course, resident macrophages in situ may also play roles in tendon repair. However, there are no reliable markers to absolutely distinguish resident macrophages from circulating macrophages, which prevents us from specifically exploring the effects of PA on resident macrophages in the present study.

Heterotopic ossification is a common complication that happens in connective tissues including tendons^38^. Previous studies have discovered that injured tendons or degenerative tendons from aged individuals would suffer the deposition of chondroid tissue and accumulated high levels of cartilage-related proteins, which severely impaired tendon structure and locomotion^7,39^. In this study, we showed that administration of PA also reduced heterotopic ossification besides inhibiting inflammation. It has been reported that in condition of favorable tendon healing, endogenous TSPCs migrated into the injured areas and correctly differentiate into tenocytes to promote tendon repair. However, when encountered with adverse external insult, such as chronic inflammation from the chemical stimulus, systemic disease, and mechanical overload, TSPCs trapped themselves into biased differentiation, such as osteogenesis or chondrogenesis, to cause undesirable heterotopic ossification^7^. In the present study, direct treatment of PA on TSPCs couldn’t inhibit osteogenic differentiation of TPSCs in vitro, indicating that reduction of heterotopic ossification by PA treatment in vivo might be attributed to indirect paracrine inhibition on TPSC osteogenic differentiation. It has been accepted that macrophage is the main immune cell at the site of tendon injury and influences the progression of tendon repair^40,41^. Moreover, studies reported that there was a frequent cross-talk among resident stromal cells, TSPCs and macrophage cells during the process of tendinopathy and tendon healing^32,42,43^. Therefore, we deduce that PA altered paracrine secretion of macrophages to suppress the osteogenic, chondrogenic differentiation of TSPCs. This deduction could be confirmed by the result that PA-treated BMDM medium effectively curbed the chondrogenic and osteogenic differentiation of TSPCs compared to the DMSO-treated BMDMs. The mTOR signaling is a crucial regulator of cell biological processes, including cell growth, metabolism, and protein synthesis. Chen et al. found that overactivation of mTOR contributed to the tendon endochondral ossification process by using in vitro experiments and conditional knockout mice^7^. Similarly, our results showed that the conditioned medium from PA-treated macrophages markedly inhibited the phosphorylation levels of mTOR in TSPCs. This finding provides an insight that modulation of inflammatory environment in tendon injury is an indispensable strategy to inhibit heterotopic ossification. Certainly, we cannot exclude the possibility that there are other secretory factors participating in the reduction of heterotopic ossification, which might need further exploration.

Leveraging technology including the nanoparticle-based delivery of small molecules provides a platform for advancing tendon therapeutics. Among various integrated nanostructured materials, the MSN has become a new generation of inorganic platforms for biomedical application^44^, because of its high loading capacity and good biocompatibility^45^. Compared with direct injection with PA solution, the delivery system exhibited the outstanding characteristic of sustained release. The sustained release property significantly reduced the required frequency of drug injection and improved the effectiveness of the treatment. In our study, the MSN@PA realized in situ release of PA, which constructed an anti-inflammation environment for TSPCs by inhibiting the secretion of inflammatory cytokines and promoting the switch into pro-reparative macrophages. Further animal experiments showed that PA accelerated the process of tendon repair and prevented the progression of heterotopic ossification with reduced injection frequency. Although the rat collagenase-induced tendinopathy model excellently presented several common pathological features of tendinopathy, including collagen disorganization, inflammation infiltration and heterotopic ossification, rodent models of tendinopathy could not fully simulate the condition of chronic tendon diseases in humans in terms of anatomy or pathology. Moreover, compared to rat or murine tendinopathy models, patients with diseased tendons in clinics have been exposed to pathological and inflammatory environments for a long time^46^. Therefore, in the near future, large animal models with similar orthograde posture and relatively longer time of pathological progression would be taken into account before clinical translation. In addition to structural assessment, functional index like mechanical testing or gait analysis would be used to evaluate tendon healing in further work.

To sum up, PA, a natural small molecule compound, modulated inflammation by promoting M2 macrophage polarization via JAK/STAT1 pathway, as well as improved macrophage paracrine secretion to suppress osteogenic and chondrogenic differentiation of TSPCs through inhibiting activation of mTOR signaling. By using a rat collagenase-induced tendinopathy model, we demonstrated that local injection of PA or local delivery of MSN@PA promoted endogenous tendon reparative capacity by inhibiting inflammation infiltration and heterotopic ossification. This study reveals that the nanoparticle-based delivery of the small molecule compound, possesses a promising translational potential for clinical therapies in tendinopathy.

## Methods

### Ethics statement

Animal experimental procedures in this study were conducted in compliance with animal welfare ethical regulations and approved by the Animal Use and Care Committee of Peking University (LA2020349). Male Sprague–Dawley rats were obtained from Weitong Lihua Experimental Animal Center (China). All rats were bred in a specific pathogen-free facility under a strict 12 h light cycle with ad libitum access to food and water, and used for experiments after 2 weeks of adaptive rearing.

### Isolation, culture and stimulation of BMDMs

Bone marrow-derived macrophages were isolated from 3 weeks Sprague–Dawley rats as previously described^47,48^. Briefly, bone marrow cells were flushed out of femurs and isolated using Lymphoprep (STEMCELL), and cultured for 1 week in RPMI 1640 medium (Solarbio) containing 10% fetal bovine serum (FBS, Thermo Fisher Scientific), 1% penicillin/streptomycin (Thermo Fisher Scientific) and 20 ng/mL recombinant rat macrophage colony-stimulating factor (M-CSF, Peprotech). The culture medium was changed once every 3 days. On day 7, adherent cells were harvested and stimulated with 100 ng/mL LPS (Sigma). After differentiation induction, BMDMs were washed with fresh medium to remove excess LPS solutions. Then, 20 μM PA (Tsbiochem) solution was added in the experimental group, whereas BMDMs in the control group were supplemented with an equal volume of dimethyl sulfoxide (DMSO, Solarbio). The sources of related reagents are described in Supplementary Table 3.

### Culture and stimulation of human THP-1-derived macrophages

Human blood monocytic cell line THP-1 was obtained from the National Infrastructure of Cell Line Resource. THP-1 monocytic cells (1 × 10^6^) were induced to differentiate into macrophages with 100 ng/mL phorbol 12-myristate 13-acetate (PMA, Sigma-Aldrich) for 24 h. The cells were cultured in complete RPMI 1640 medium without PMA and stimulated with 100 ng/mL LPS. Then, the cells were washed with fresh medium to remove any excess LPS and treated with 20 μM PA or an equal volume of PBS.

### Isolation and culture of TSPCs

Primary TSPCs were isolated from 6–8 weeks rats according to the established procedure^49^. The harvested tendon was minced and digested completely with 3 mg/mL collagenase type I (Thermo Fisher Scientific) and 4 mg/mL dispase (Roche) at 37 °C for 1 h. After passing through a 70 μm strainer, single-cell suspensions were cultured in low-glucose DulbeccoA’s modified Eagle’s medium (DMEM, Hyclone) supplemented with 15% fetal bovine serum (FBS, Thermo Fisher Scientific), 2 mM L-glutamine (Thermo Fisher Scientific), and 100 U/mL penicillin/streptomycin (Thermo Fisher Scientific) in an incubator at 37 °C with 5% CO_2_. When the cells reached 80 – 90% confluency, they were passaged. TSPCs at passage 2–4 were used in further experiments.

### Multipotent differentiation of TSPCs in BMDM-conditioned medium

Regarding the differentiation experiment, TSPCs were cultured in 12-well plates (50,000 cells/well). Osteogenic and chondrogenic differentiation were induced by a corresponding differentiation medium and supplemented with the collected supernatant from BMDM-conditioned medium at a ratio of 1:1. The osteogenic medium consisted of a growth medium supplemented with 10 nM dexamethasone (Sigma-Aldrich), 0.05 mM l-ascorbic acid 2-phosphate (Sigma-Aldrich), and 5 mM β-glycerol phosphate (APEXBIO). After culture in an osteogenic medium for 14 days, TSPCs were stained with Alizarin Red S (ARS, Sigma-Aldrich) to evaluate osteogenic differentiation capacity. The chondrogenic medium contained a growth medium supplemented with 2 mM sodium pyruvate (Hyclone), 1% Insulin-Transferrin-Selenium (Thermo Fisher Scientific), 50 μg/mL ascorbic phosphate (Sigma-Aldrich), 10 nM dexamethasone, and 10 ng/mL transforming growth factor-β (TGF-β, Peprotech). After culture in a chondrogenic medium for 14 days, TSPCs were stained with Alcian blue.

### Live/dead staining

Live/dead staining was carried out according to the manufacturer’s protocol. Briefly, cells were incubated 30 min in PBS containing 2 μM calcein-AM (Solarbio) and 4.5 μM Propidium iodide, (PI, Solarbio). Fluorescence images were acquired with a confocal fluorescence microscope (TCS SP8, Leica).

### Quantitative real-time polymerase chain reaction

Total RNA was isolated from BMDMs, THP-1-derived macrophages, or TPSCs using a Trizol reagent (Thermo Fisher Scientific) according to the manufacturer’s instruction. The concentration of purified total RNA was then determined using a NanoDrop spectrophotometer (Thermo Fisher Scientific, Wilmington, DE). Next, two micrograms of total RNA were reverse-transcribed to complementary DNA (cDNA) using Prime Script RT Reagent Kit (Takara) following the manufacturer’s protocol. Quantitative RT-PCR was performed using gene-specific primers and SYBR Green (Invitrogen), finally run on 7900HT Fast Time PCR. Primer sequences were listed in Supplementary Tables 4, 5.

### Western blotting

Total proteins in cell lysates were harvested by RIPA Buffer (Thermo Fisher Scientific) with Protease/Phosphatase Inhibitor Cocktail (Thermo Fisher Scientific). The protein concentration was determined using the Pierce BCA protein assay kit (Thermo Fisher Scientific). Cell lysate proteins were separated by 10% SDS-PAGE gels, and then transferred to polyvinylidene difluoride membranes and blocked in 5% nonfat milk. The membranes were incubated with anti-CD206, anti-iNOS, anti-IL-6, anti-ARG-1, anti-JAK-1, anti-p-STAT1, anti-STAT1, anti-OSX, anti-OCN, anti-p-mTOR, anti-mTOR at a dilution ratio of 1:1000 and incubated with anti-ACTIN, anti-VINCULIN, anti-TUBULIN, anti-GAPDH at a dilution ratio of 1:3000 overnight at 4 °C. After washed three times in TBS with 0.1% Tween-20, membranes were probed with appropriate secondary antibodies at a dilution ratio of 1:5000 for 1 h at room temperature. The membranes were washed twice in TBS with 0.1% Tween-20 and imaged. The relative density was measured using ImageJ 1.53k software (Wayne Rasband). All blots or gels derived from the same experiment and were processed in parallel. Detailed information of primary and secondary antibodies was listed in Supplementary Table 6.

### Immunocytochemistry

Cells were seeded onto 24-well plates with cell culture slides. After the required time, the cells were fixed in 4% paraformaldehyde for 15 min at room temperature. If permeabilization, the samples were permeabilized with 0.2% Triton-X, then blocked with TBST containing 5% normal donkey serum and incubated with corresponding primary antibodies at a dilution ratio of 1:100 for 1 h at room temperature. Next, cells were incubated with Alexa Fluor 488- and Alexa Fluor 594- conjugated secondary antibodies (ZSGB-BIO, 1:300) diluted in TBST for 1 h at room temperature. Finally, nuclei were stained with DAPI (ZSGB-BIO) and confocal microscopic images were acquired with a Zeiss laser-scanning microscope 710 or a Leica TCS SP8 STED confocal microscope.

### Synthesis of MSNs, rhodamine-loaded MSNs and MSN@PA

Preparation of MSNs was first carried out according to reported literature with slight modification^26,50^. Briefly, 48 mL of 25 wt % hexadecyl trimethyl ammonium chloride (CTAC) solution and 0.36 g of triethanolamine (TEA) were added to 72 mL of deionized water and stirred gently at 60 °C in an oil bath under a magnetic stirring for at least 1 h. After stirring, 40 mL of (20 v/v %) tetraethyl orthosilicate (TEOS) in cyclohexane was carefully added to the surface of water-CTAC-TEA solution and kept at 60 °C in an oil bath under a magnetic stirring for 24 h. The products were centrifuged at 25,000 g for 30 min and washed three times with ethanol to remove the residual reactants. The collected products were extracted with a 10 v/v % hydrochloric acid-methanol solution at 60 °C for 6 h twice to remove the template.

In order to trace nanomedicine distribution, click-chemistry between Rhodamine B (RhB, Sigma) and MSNs need to be achieved. First, amine silane was grafted onto the MSN to provide a reactive surface for covalent conjugation with PEG-derivatives. Simply, 50 mg of MSNs were dispersed in 25 mL of ethanol with 0.5 mL of ammonium hydroxide (28–30%, Sigma) as a catalyst. Then, 1 mL of 3- aminopropyl triethoxysilane (APTES, Sigma) was dropwise added, stirring at 25 °C for 24 h. After the reaction, MSN-NH_2_ was obtained by centrifugation and washing with ethanol to remove residual APTES and catalyst. Then, the purified 50 mg MSN-NH_2_ was incubated in 25 mL of ethanol containing 1.25 mg of a fluorescent dye of RhB for 24 h at 25 °C, followed by centrifuging and repeated washing with ethanol to remove physisorbed rhodamine B molecules from the exterior surface of the material. This reaction also depended on the click-chemistry between an amine group and an isothiocyanate group. The collected RhB-labeled MSNs (RhB-MSNs) were washed and kept in ethanol for further experiments.

PA was loaded into the pores of MSNs and RhB-MSNs by rotary evaporation. At first, 3 mg of PA, 7 mg of MSNs and RhB-labeled MSNs and 1 mL of methanol were mixed. Then, methanol was slowly evaporated under nitrogen purging. Finally, the dry powder of MSN@PA and RhB-MSN@PA was obtained.

### Scanning electron microscopy

The surface morphologies of nanoparticles and neo-tissues were investigated using SEM (Hitachi S-4800, Japan). The nanoparticle specimens were uncoated for observation. The neo-tissues were pre-fixed in 2.5% glutaraldehyde in PBS (pH 7.4) at 4 °C for 12 h and washed three times with PBS. Then, they were dehydrated in a graded series of ethanol (50–100%), critical-point dried, and sputter-coated with gold for 2 min at 20 mA.

### Transmission electron microscopy

TEM specimens were dispersed in ethanol and then transferred to a copper grid. The morphology of the nanomaterials was then observed (TEM, Tecnai F20, FEI, USA) at 100 kV. Nanoparticle diameter was measured from the TEM images by marking the nanomaterials individually in Image-Pro Plus 6.0 software.

### Dynamic light scattering

DLS was applied to determine the size distribution of MSNs and MSN@PA. Briefly, 0.1 mg/mL samples were prepared in PBS, and particle sizes were measured using a Zetasizer Pro (Malvern Instruments Ltd, UK).

### Fourier transform infrared spectroscopy

Chemical analysis of PA, MSNs and MSN@PA was revealed by FTIR spectroscopy. FTIR spectra were performed on a liquid nitrogen-cooled spectrometer (Thermo Fisher Scientific, Nicolet iN10) equipped with a diamond compression cell to flatten them to a thickness suitable for FTIR transmission measurements.

### Thermal gravimetric analysis

Thermal gravimetric analysis (TGA) was performed under a nitrogen atmosphere using a thermogravimetric analyzer (Mettler Toledo), with the temperature range from room temperature to 800 °C at a heating rate of 10 °C/min. 3 mg of each sample was used for the TGA measurement.

### Drug release experiments

The drug release curve was constructed by measuring the amounts of PA released from drug-loaded MSNs at various time intervals. Briefly, 10 mL of nanoparticle suspension in PBS (1 mg/mL) were infused in a dialysis bag (12–14 kDa), and they were immersed in 10 mL of PBS at 37 °C for 21 days. At predetermined time intervals, 0.5 mL release buffer was collected for measurement, and the same volume of fresh buffer was replenished. The concentration of released PA was determined with a UV–Vis absorption spectrometer (Agilent, Cary 60). The absorbance of PA at 227 nm was used to determine the concentration of the drug. The cumulative amount of PA released at a particular measurement time was determined by summing the amount measured at that time and the cumulative amount measured at the last measurement.

### Biodistribution of MSN@PA

To visualize the distribution of nanoparticles in the injured tendon after injection and confirm the retention of nanoparticles, rats were injected with RhB-MSN@PA, and the in vivo nanoparticle distribution was analyzed with a fluorescence imaging system at different time points (0, 5, 7 days after injection). These processes were subjected to ex vivo imaging and analyzed by an IVIS Spectrum In Vivo Imaging System (PerkinElmer).

### A rat tendinopathy model

To explore the therapeutic effect of PA in tendinopathy, we establish a rat tendinopathy model^51^. Eighteen male Sprague–Dawley rats (8–10 weeks, body weight of ~300 g) were randomly distributed into three groups: Sham (*n* = 6), PBS (*n* = 6), and PA (*n* = 6). During anesthetization, 100 μL of collagenase type I (50 mg/mL) was introduced into the rat’s right Achilles tendon tissues, except the sham group. After 1 weeks, all groups were treated differently. In the PBS group, the rats were injected with 100 μL PBS three times a week in the Achilles tendon region. In the PA group, the rats were injected with 100 μL PA (2 mg/kg) thrice a week. During the treatment period, all rats were allowed free cage activities. After one and 5 weeks, respectively, the rats were euthanized via CO_2_ asphyxiation and their Achilles tendons were harvested for further experiments.

We established a rat tendinopathy model to detect sustained release and treatment efficacy. Thirty male Sprague–Dawley rats (8–10 weeks, body weight of ~300 g) were randomly distributed into the five groups: sham (*n* = 6), PBS (*n* = 6), PA (*n* = 6), MSNs (*n* = 6) and MSN@PA (*n* = 6) groups. During anesthetization, 100 μL of collagenase type I (50 mg/mL) was introduced into the rat’s right Achilles tendon tissues, except the sham group. After 1 week, the rat in the sham and PBS group were injected with 100 μL PBS twice a week in the Achilles tendons. The rats in the PA group were injected with 100 μL PA solutions (2 mg/kg) twice a week. The rats in other groups were injected with the MSN and MSN@PA. During the treatment period, all rats were allowed free cage activities. Five weeks after operation, the rats were euthanized via CO_2_ asphyxiation and their Achilles tendons were harvested for further study.

### Histological, immunohistochemical and immunofluorescent stainings

Tissue specimens were fixed in 4% paraformaldehyde, washed with running water, dehydrated in a graded ethanol series, vitrified with dimethylbenzene, and embedded in paraffin. Histological sections with 5 μm in thickness were prepared using a microtome. To examine the microstructure of soft tissues, HE, Masson’s trichrome, Alcian blue and Safranine O staining were performed according to standard procedures. Sirius red staining was performed using Sirius red and picric acid, and then sections were visualized under polarized light. All slides were interpreted using the semi-quantitative grading scale of Movin score^20^, assessing various aspects of tendon tissues. Fiber structure, fiber arrangement, nuclei rounding, regional variations of cellularity, decreased collagen staining, hyalinization, and the presence of acute inflammatory characteristics were included in the scale. Each slice was given a score between 0 and 3. In this scoring system, the values are assigned as follows: 0, normal; 1, slightly abnormal; 2, abnormal and 3, markedly abnormal. As the degeneration progressed, the score increased. The protein expression in the tissues was examined using immunochemistry or immunofluorescence. For immunohistochemical staining, tissue sections were deparaffinized, blocked and incubated with anti-IL6, and anti-TNF-α at a dilution ratio of 1:100 overnight at 4 °C. The slides were stained with enhanced enzyme-labeled goat anti-mouse IgG polymer (ZSGB-BIO) and enhanced enzyme-labeled goat anti-rabbit IgG polymer (ZSGB-BIO) at next day, and visualized with a DAB peroxidase substrate kit (ZSGB-BIO). For immunofluorescent staining, sections were incubated with anti-CD68, anti-CD206, anti-iNOS, anti-FMOD, anti-TNC, Anti-TNMD, anti-Aggrecan, anti-COL II, anti-p-STAT1, anti-p-mTOR at a dilution ratio of 1:100. Next, the sections were incubated with fluorescein isothiocyanate-conjugated or tetramethylrhodamine isothiocyanate-conjugated secondary antibodies (ZSGB-BIO, 1:300). Nuclei were counterstained with 4′,6-diamidino-2-phenylindole. Histological image acquisition was performed with a Nikon microscope. Confocal microscopic images were acquired with a Zeiss laser-scanning microscope 710 or a Leica TCS SP8 STED confocal microscope. The detailed information of used antibodies was shown in Supplementary Table 6.

### Micro-CT scanning

The rat hindlimbs were fixed in 10% neutral buffered formalin. Then, rat hindlimbs were scanned by a Skyscan 1174 micro-CT system (Bruker, Belgium). The scanner was set at a voltage of 80 kV and a resolution of 20.74 μm per pixel. The acquired axial images were imported into a NRecon and CTvox software for visualization and analysis. 3-D reconstruction was performed for evaluating heterotopic ossification. We defined the regions of interest and calculated bone volume (BV) parameter of the interested region for each specimen by using CTAn software. The detailed information of the used software is shown in Supplementary Table 7.

### Safety aspects of nanomaterials

PBS, MSNs, PA, and MSN@PA were administered through local injection of rats at a dose of 2 mg kg^−1^ twice. The internal organs (heart, liver, lung, and kidney) were collected 5 weeks post-injection for histological analysis.

### Statistical analysis

All experiments were performed at least three times. The quantitative data were presented as means ± SD. An unpaired t-test was performed to assess whether statistical differences existed between groups. Multiple comparisons were performed with a one-way analysis of variance (ANOVA) and Tukey’s post-test. *p* values < 0.05 were considered statistically significant. The significance level is presented as *p < 0.05, **p < 0.01, ***p < 0.001 and ****p < 0.0001. Statistical analyses were performed using GraphPad Prism® software (version 9.0, GraphPad Software Inc, California, USA).

### Reporting summary

Further information on research design is available in the Nature Research Reporting Summary linked to this article.

## Supplementary information

Revised Supporting information-Clean version

## Data Availability

All data needed to evaluate or reproduce the conclusions in the paper are present in the paper and/or the Supplementary Materials. All data generated during and/or analyzed during the current study are available from the corresponding author upon reasonable request.
