# Author Correction: Parishin A-loaded mesoporous silica nanoparticles modulate macrophage polarization to attenuate tendinopathy

**DOI:** 10.1038/s41536-026-00480-z

**Published:** 2026-05-28

**Authors:** Lisha Zhu, Yu Wang, Shanshan Jin, Yuting Niu, Min Yu, Zixin Li, Liyuan Chen, Xiaolan Wu, Chengye Ding, Tianhao Wu, Xinmeng Shi, Yixin Zhang, Dan Luo, Yan Liu

**Affiliations:** 1 Laboratory of Biomimetic Nanomaterials, Department of Orthodontics, Peking University School and Hospital of Stomatology, Beijing, China; 2 Beijing Institute of Nanoenergy and Nanosystems, Chinese Academy of Sciences, Beijing, China; 3 National Center for Stomatology & National Clinical Research Center for Oral Diseases & National Engineering Laboratory for Digital and Material Technology of Stomatology & Beijing Key Laboratory of Digital Stomatology & Research Center of Engineering and Technology for Computerized Dentistry Ministry of Health & NMPA Key Laboratory for Dental Materials, Beijing, China; 4 Central Laboratory, Peking University School and Hospital of Stomatology, Beijing, China; 5 Department of Prosthodontics, Peking University School and Hospital of Stomatology, Beijing, China

**Keywords:** Drug delivery, Diseases

Correction to: *npj Regenerative Medicine* 10.1038/s41536-023-00289-0, published online 10 March 2023

In the original version of this article, Figs. 2, 5, and Supplementary Fig. 1 appeared incorrectly. In Fig. 2i, the iNOS PA 5 weeks and CD206 PBS 5 weeks panels were incorrect. In Fig. 5b, the HE PA panel, Masson PA magnified panel, and Masson MSN@PA magnified panel were incorrectly positioned. In Supplementary Fig. 1c, the 0 µM panel was incorrect. The original article has been corrected.

**Corrected Figure 2** 
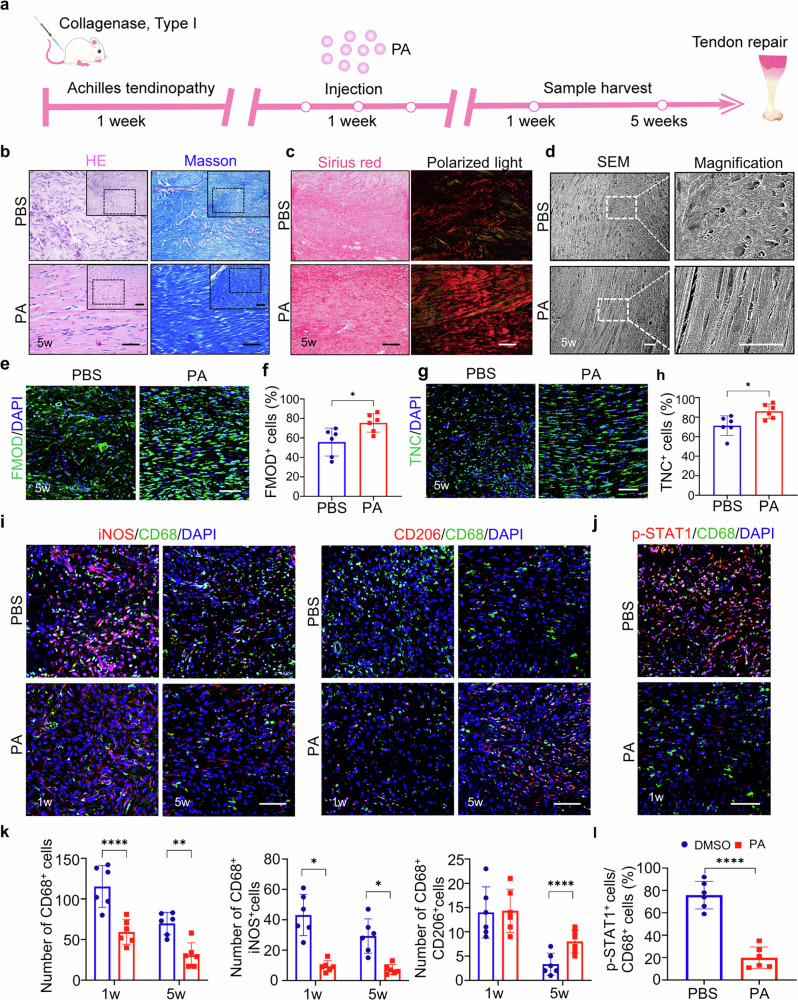


**Incorrect Figure 2** 
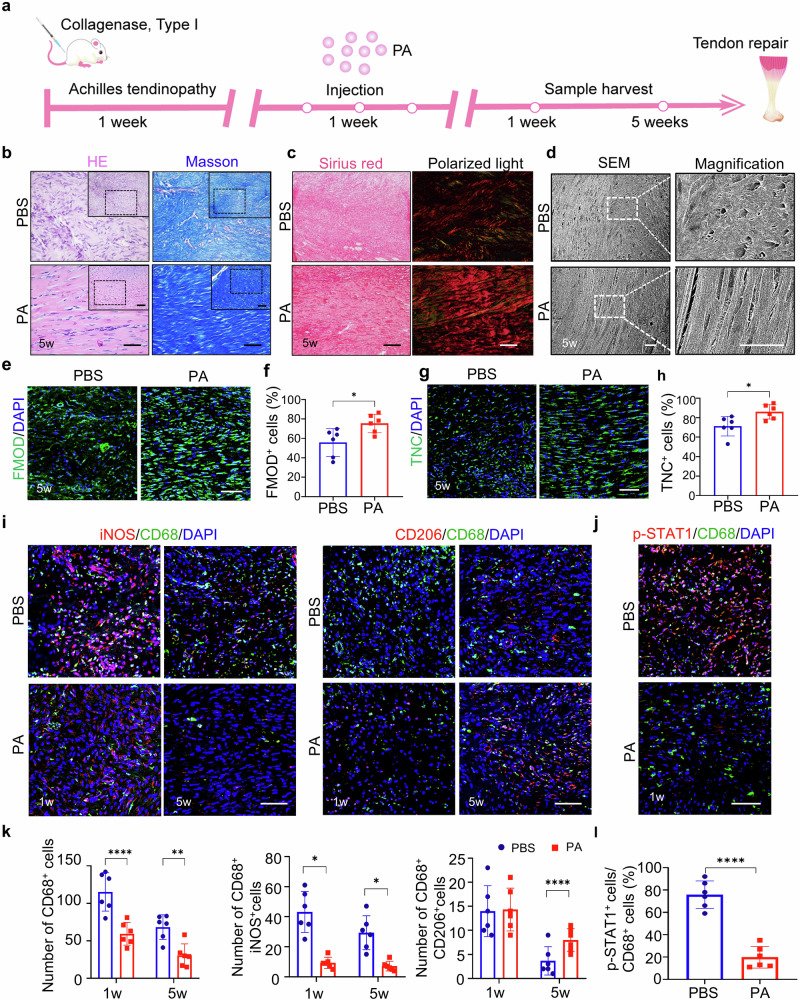


**Corrected Figure 5** 
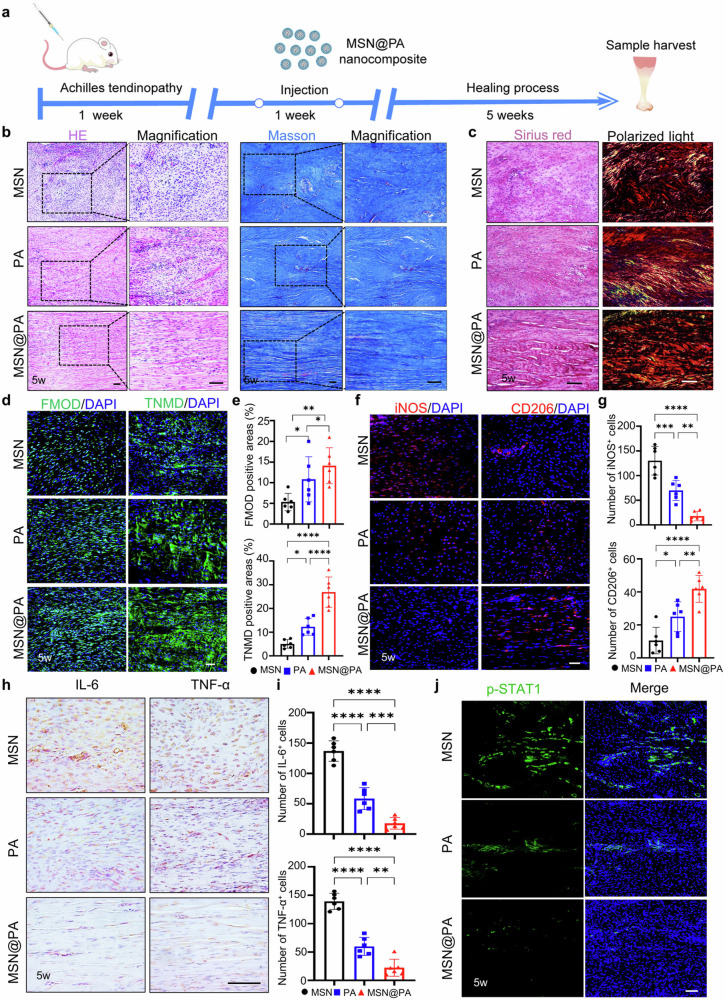


**Incorrect Figure 5** 
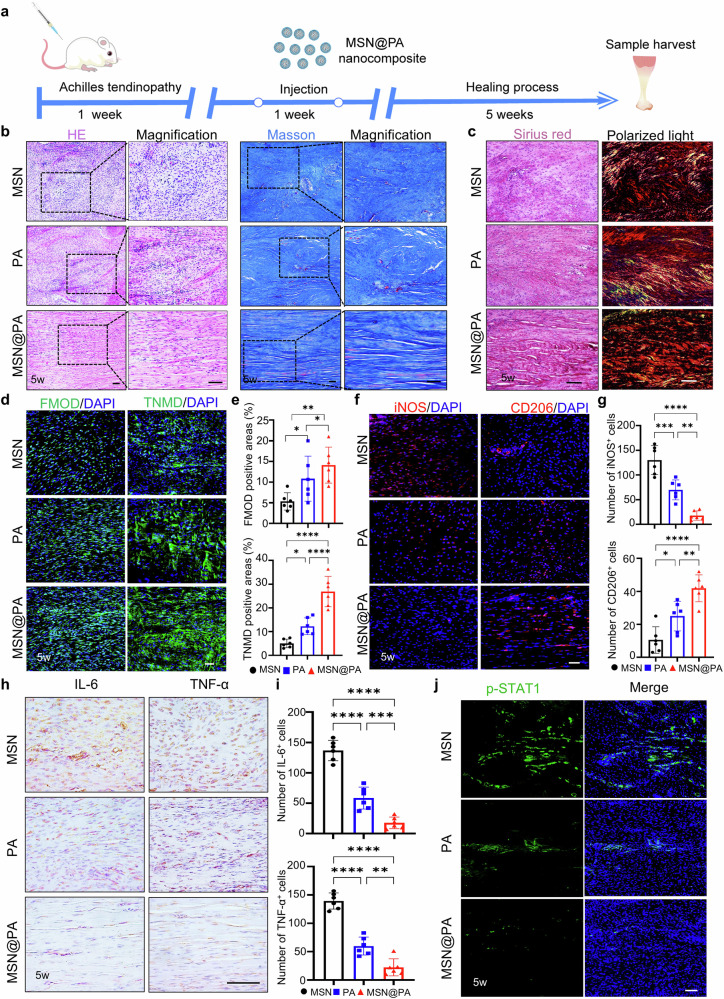


## Supplementary information

Corrected supporting information

