# Supplementary material for: Author Correction: Parishin A-loaded mesoporous silica nanoparticles modulate macrophage polarization to attenuate tendinopathy
Source: NPJ Regen Med. 2026 May 28;11:25. doi: 10.1038/s41536-026-00480-z (PMC13219431; doi:10.1038/s41536-026-00480-z)
Supplement: Supplementary file 1 — Corrected supporting information [file 41536_2026_480_MOESM1_ESM.pdf]

Parishin A-loaded Mesoporous Silica Nanoparticles Modulate Macrophage Polarization to Attenuate Tendinopathy

Lisha Zhu[1, 2, 5], Yu Wang[1, 5], Shanshan Jin[1, 5], Yuting Niu[3, 4], Min Yu[4], Zixin Li[1], Liyuan Chen[1], Xiaolan Wu[1], Chengye Ding[1], Tianhao Wu[1], Xinmeng Shi[1], Yixin Zhang[1], Dan Luo[2*], Yan Liu[1*]

[1]Laboratory of Biomimetic Nanomaterials, Department of Orthodontics, Peking University School and Hospital of Stomatology & National Center for Stomatology & National Clinical Research Center for Oral Diseases & National Engineering Laboratory for Digital and Material Technology of Stomatology & Beijing Key Laboratory of Digital Stomatology & Research Center of Engineering and Technology for Computerized Dentistry Ministry of Health & NMPA Key Laboratory for Dental Materials, Beijing 100081, China

[2]Beijing Institute of Nanoenergy and Nanosystems, Chinese Academy of Sciences, Beijing 101400, China

[3]Central Laboratory, Peking University School and Hospital of Stomatology & National Center for Stomatology & National Clinical Research Center for Oral Diseases & National Engineering Laboratory for Digital and Material Technology of Stomatology & Beijing Key Laboratory of Digital Stomatology & Research Center of Engineering and Technology for Computerized Dentistry Ministry of Health & NMPA Key Laboratory for Dental Materials, Beijing 100081, China

[4]Department of Prosthodontics, Peking University School and Hospital of Stomatology & National Center for Stomatology & National Clinical Research Center for Oral Diseases & National Engineering Laboratory for Digital and Material Technology of Stomatology & Beijing Key Laboratory of Digital Stomatology & Research Center of Engineering and Technology for Computerized Dentistry Ministry of Health & NMPA

Key Laboratory for Dental Materials, Beijing 100081, China

[5]These authors contributed equally: Lisha Zhu, Yu Wang, Shanshan Jin.

[*]Corresponding author. Email: luodan@binn.cas.cn (D.L.) orthoyan@bjmu.edu.cn (Y.L.)

Table of contents

Supplementary Figure 1. Chemical structure and effective concentrations of PA.

Supplementary Figure 2. Semi-quantification of Western blots in Figure 1b.

Supplementary Figure 3. The regulatory effect of PA on human THP-1 derived macrophage polarization and the mechanisms.

Supplementary Figure 4. PA alleviates excessive inflammatory response in the early stage of tendon healing.

Supplementary Figure 5. PA prevents tendon heterotopic ossification.

Supplementary Figure 6. Sustained-release performance and *in vitro* cytotoxicity of MSN.

Supplementary Figure 7. The biological safety of MSN@PA.

Supplementary Figure 8. Original scans of the blots in Fig. 1b, 1e, 3k, 3l.

Supplementary Figure 9. Original scans of the blots in Supplementary Fig. 1b, 3b, 3d.

Supplementary Table 1. Modified Movin score of tendons from the PBS and PA groups.

Supplementary Table 2. Modified Movin score of tendons from the MSN, PA and MSN@PA groups.

Supplementary Table 3. List of reagents or resources used in the study.

Supplementary Table 4. List of rat primers used in the study.

Supplementary Table 5. List of human primers used in the study.

Supplementary Table 6. List of primary and secondary antibodies used in the study.

Supplementary Table 7. Software.

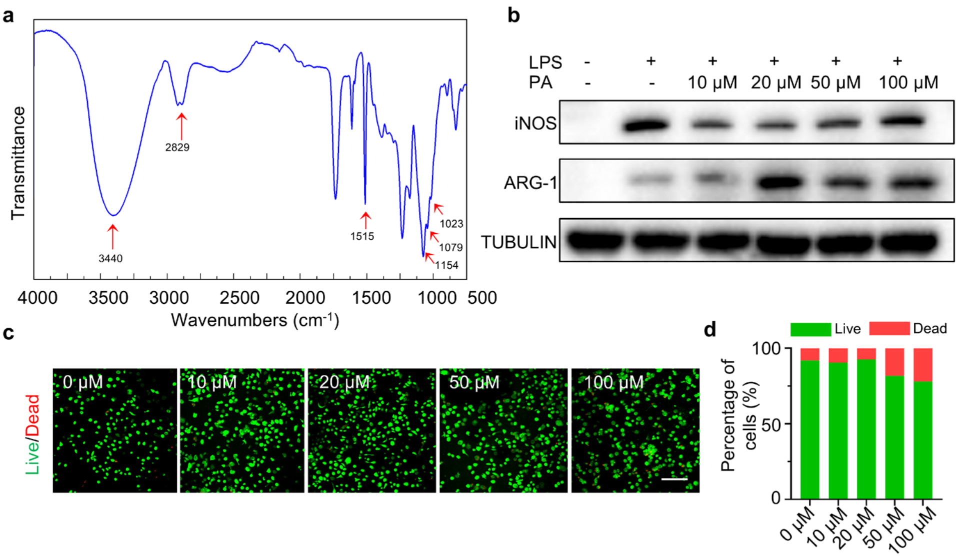

Supplementary Figure 1. Chemical structure and effective concentrations of PA. **a** Fourier translation infrared spectroscopy (FTIR) spectra of PA. **b** Western blotting of iNOS and ARG-1 in and PA-treated human THP-1 cells at the concentration of 0, 10 μM, 20 μM, 50 μM and 100 μM. Scale bar, 200 μm. **c** Live/Dead staining of BMDMs treated with dose-dependent PA for 48 h. **d** Semi-quantification of (**c**).

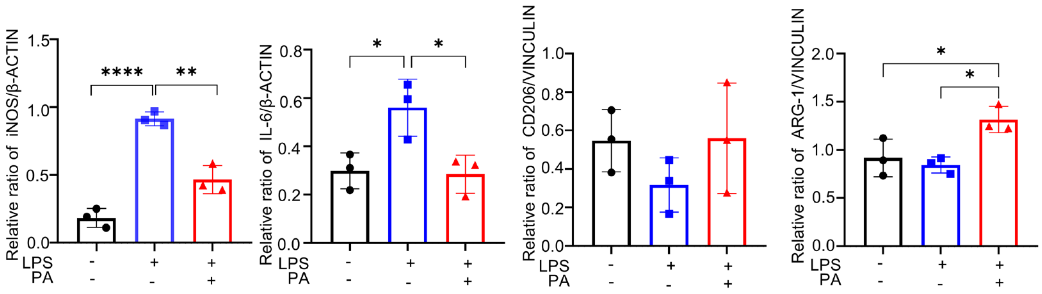

Supplementary Figure 2. Semi-quantification of Western blots in Figure 1b. Data are presented as mean ± SD. * $p < 0.05$, ** $p < 0.01$ and **** $p < 0.0001$, n =3.

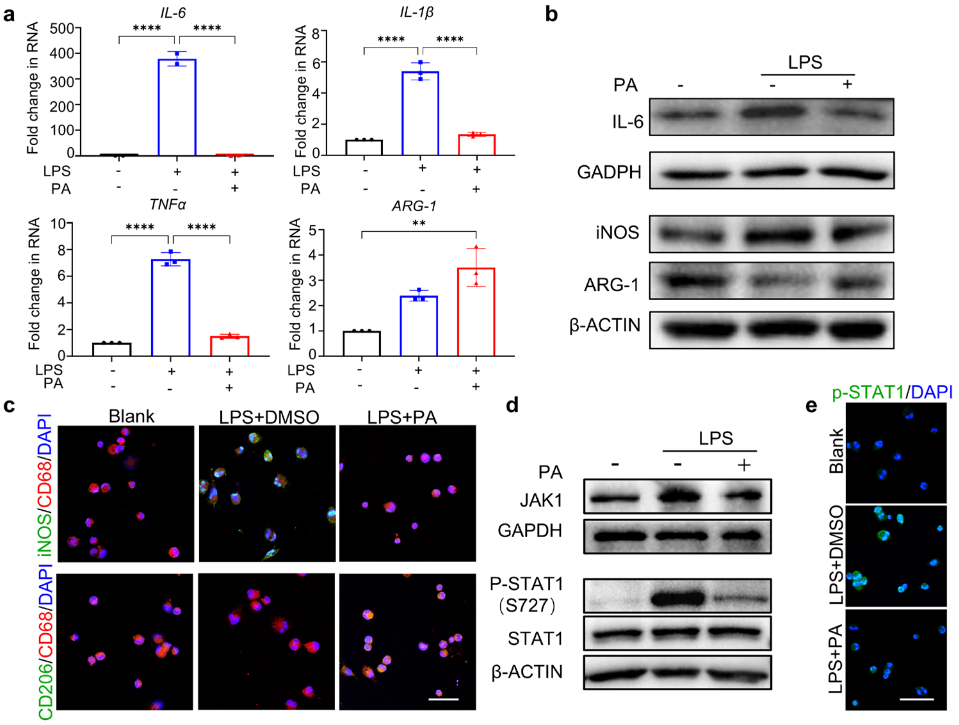

Supplementary Figure 3. The regulatory effect of PA on human THP-1 derived macrophage polarization and the mechanisms. a RT-PCR of *IL-6*, *IL-1β*, *TNF-α* and *ARG-1* expression in DMSO- and PA-treated human THP-1 cells under LPS stimulation. **b** Western blotting of iNOS, IL-6, and ARG-1 in DMSO- and PA-treated THP-1 cells under LPS stimulation. **c** Co-immunofluorescence staining of iNOS/CD68 and CD206/CD68 in unstimulated THP-1 cells and DMSO, PA-treated THP-1 cells under LPS stimulation. Scale bar, 50 μm. **d** Western blotting of total JAK1, phosphorylation of STAT1 and total STAT1 in DMSO- and PA-treated THP-1 cells under LPS stimulation. **e** Immunofluorescence staining of p-STAT1 in DMSO- and PA-treated THP-1 cells under LPS stimulation. Scale bar, 100 μm. Data are presented as mean ± SD. ** $p < 0.01$ and **** $p < 0.0001$, $n = 3$.

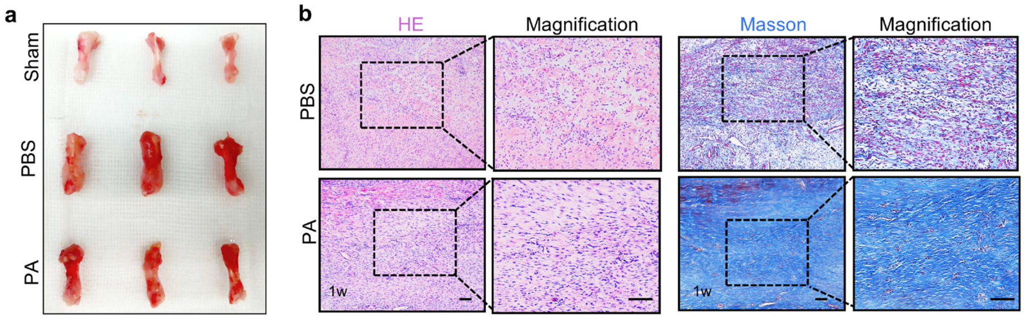

Supplementary Figure 4. PA alleviates excessive inflammatory response in the early stage of tendon healing. a Gross view of tendon on 1 week of post-injection. **b** HE and Masson's trichrome stainings of neo-tendons from PBS and PA groups at 1 week. Scale bar, 100 μm.

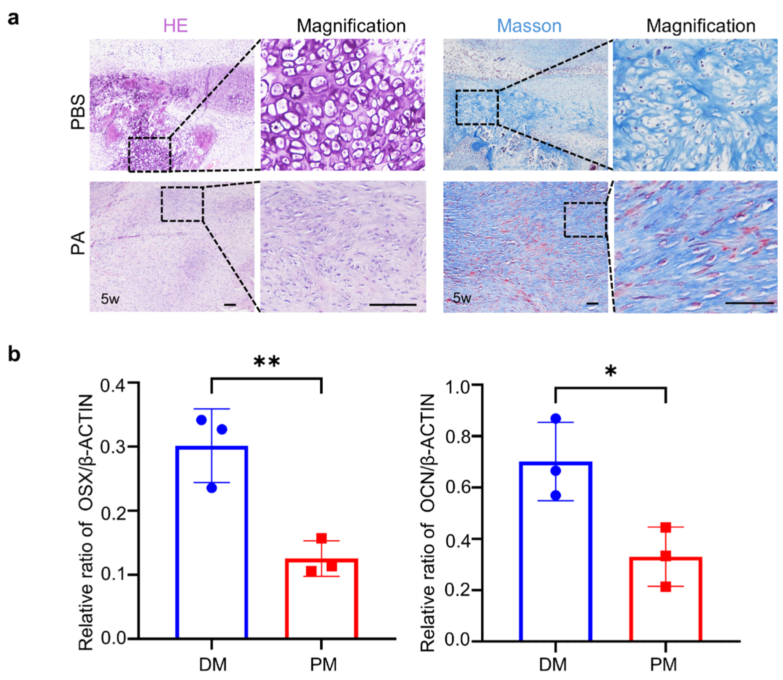

Supplementary Figure 5. PA prevents tendon heterotopic ossification. a HE and Masson's trichrome stainings of region of heterotopic ossification from PBS and PA groups at 5 weeks. Scale bar, 100 μm. **b** Semi-quantification of Western blots in Figure 3k. Data are presented as mean ± SD. * $p < 0.05$ and ** $p < 0.01$, n =3.

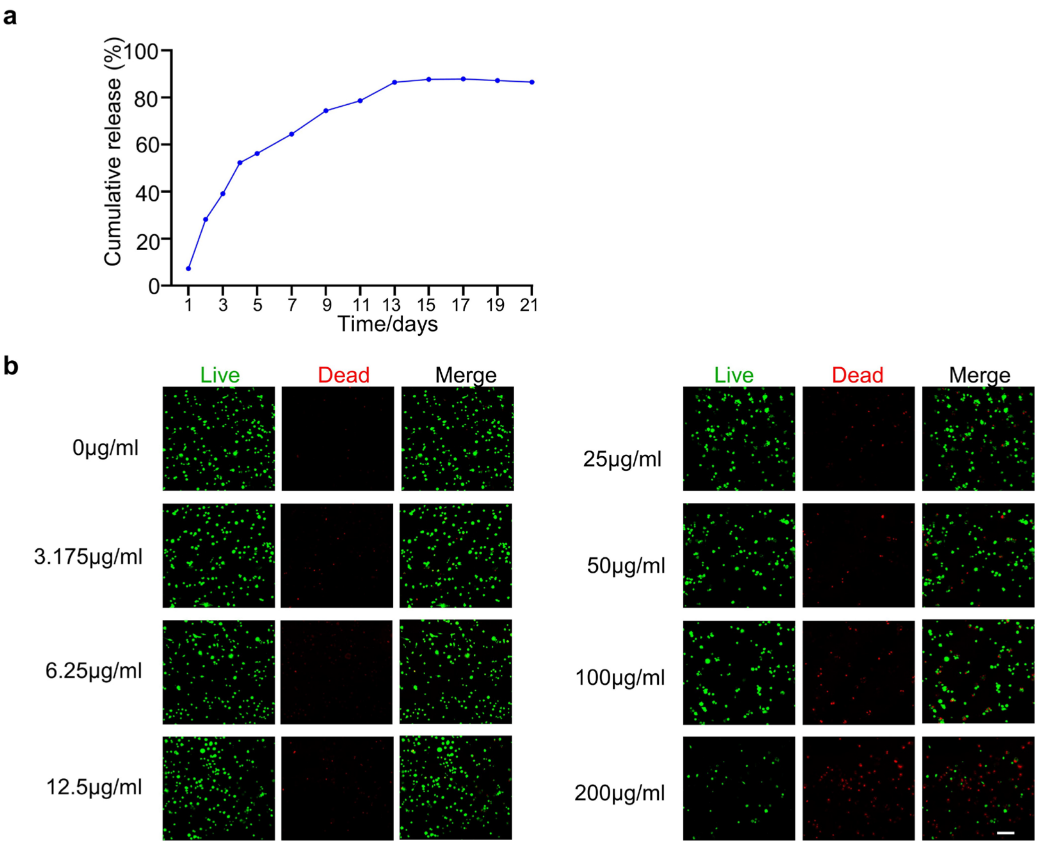

Supplementary Figure 6. Sustained-release performance and *in vitro* cytotoxicity of MSN. a Cumulative release concentration curve of PA from MSNs in PBS solution. **b** Live/Dead staining of BMDMs treated with dose-dependent the MSNs for 24 h. Scale bar, 200 μm.

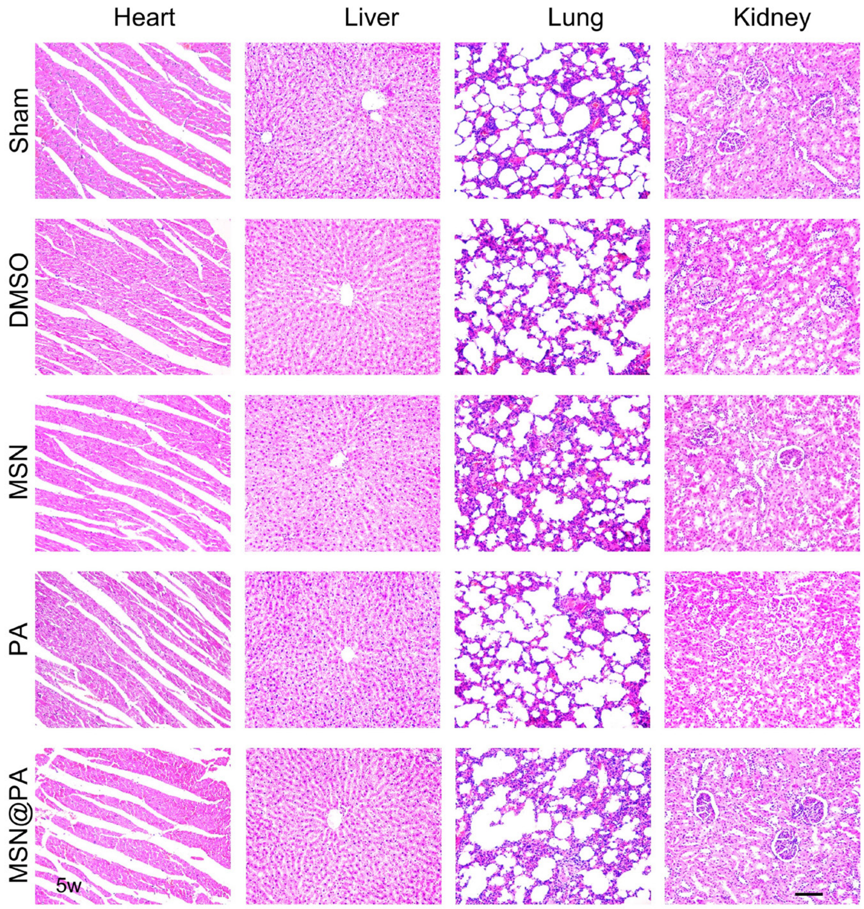

Supplementary Figure 7. The biological safety of MSN@PA. HE staining results of representative tissue sections of rats (heart, liver, lung, and kidney) after injection of MSN, PA and MSN@PA. Scale bar, 100 μm.

Fig.1b

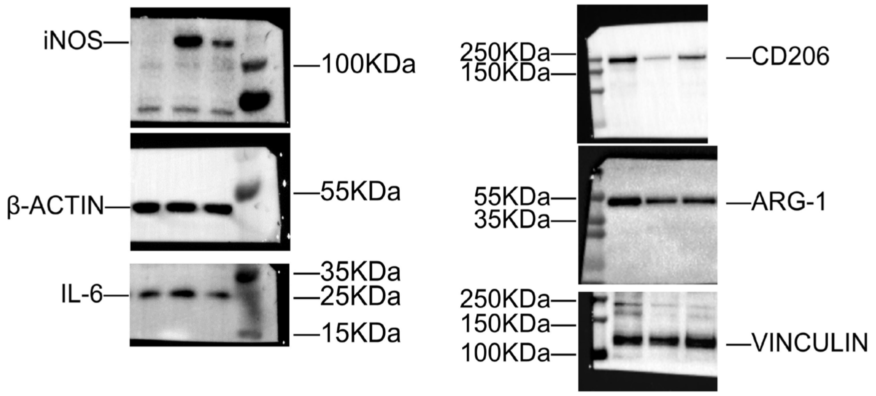

Fig.1e

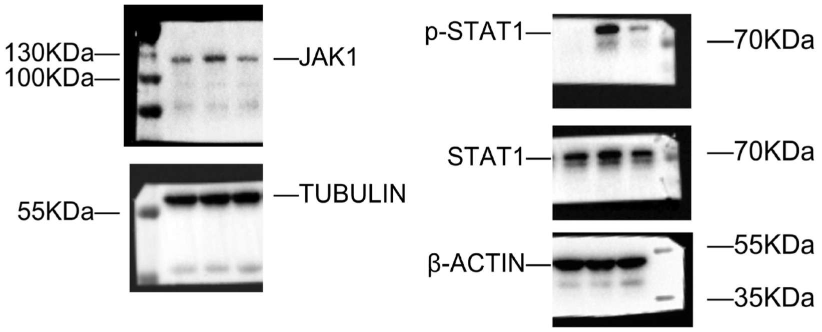

Fig.3k

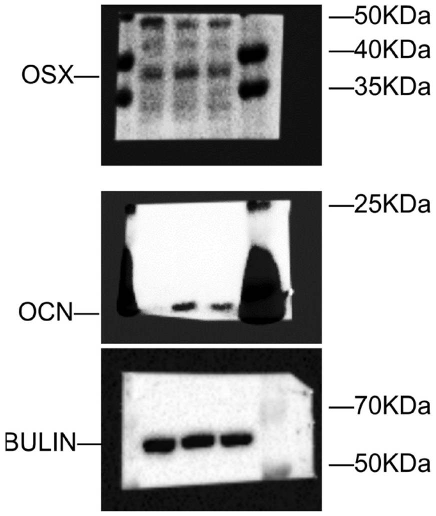

Fig.3l

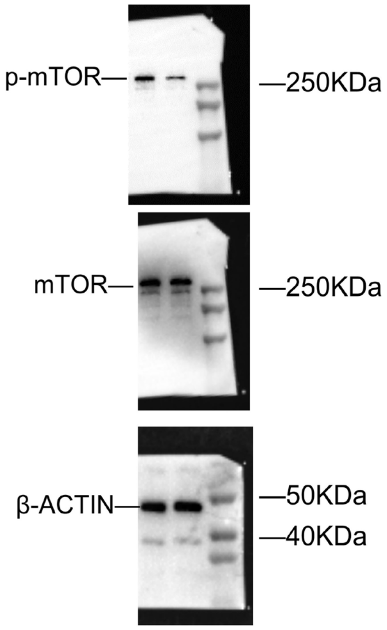

Supplementary Figure 8. Original scans of the blots in Fig. 1b, 1e, 3k, 3l.

Supplementary Fig.1b

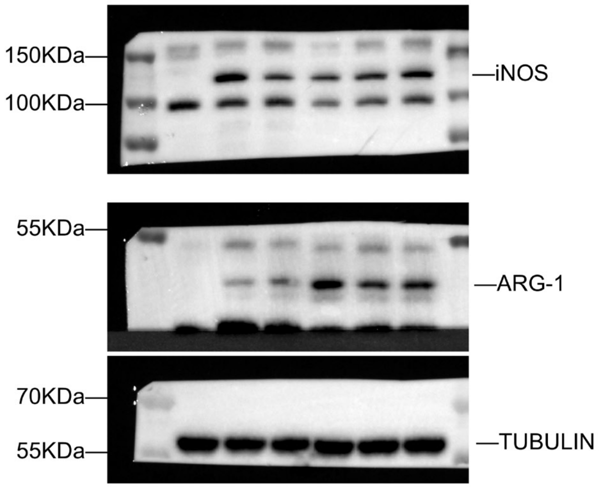

Supplementary Fig.3b

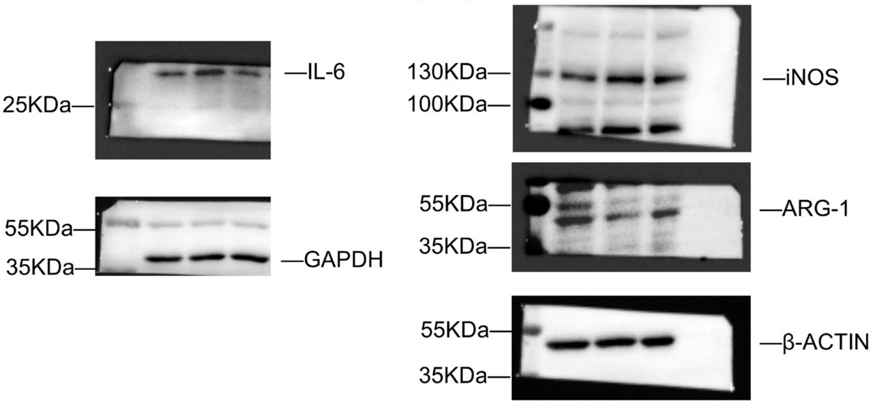

Supplementary Fig.3d

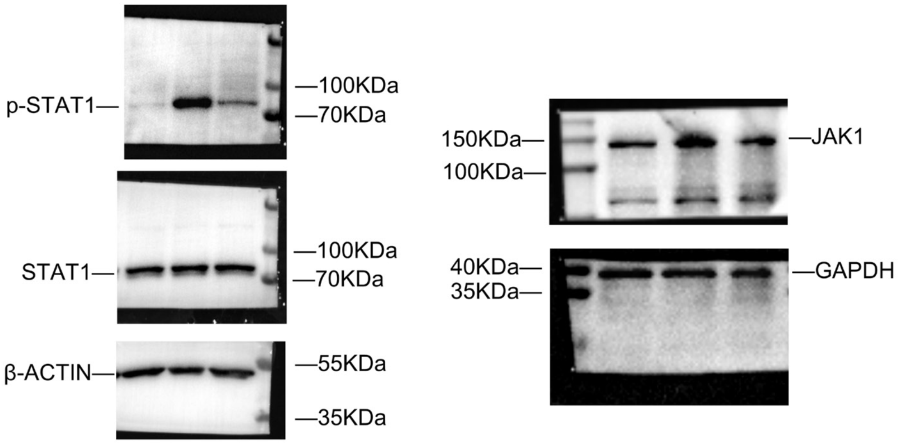

Supplementary Figure 9. Original scans of the blots in Supplementary Fig. 1b, 3b, 3d.

Supplementary Table 1. Modified Movin score of tendons from the PBS and PA groups.

Variable	PBS group (*n* = 6)	PA group (*n*=6)
Fiber structure	2.667 ± 0.516	1.500 ± 0.547**
Fiber arrangement	2.883 ± 0.408	1.667 ± 0.516**
Rounding of nuclei	2.500 ± 0.547	1.333 ± 0.516**
Regional variations of cellularity	2.667 ± 0.516	1.667 ± 0.816
Increase in vascularity	2.500 ± 0.547	1.667 ± 0.516**
Decreased collagen stainability	2.667 ± 0.516	1.167 ± 0.408**

Note: Values are Mean ± SD; **: $P < 0.01$ *versus* PBS group.

Supplementary Table 2. Modified Movin score of tendons from the MSN, PA and MSN@PA groups.

Variable	MSN group (*n*=6)	PA group (*n*=6)	MSN@PA group (*n*=6)
Fiber structure	2.883 ± 0.408	1.667 ± 0.516**	1.333 ± 0.516***
Fiber arrangement	2.883 ± 0.408	2.000 ± 0.633	1.500 ± 0.837**
Rounding of nuclei	2.667 ± 0.516	1.667 ± 0.816	1.500 ± 0.837*
Regional variations of cellularity	2.500 ± 0.837	1.667 ± 0.816	1.000 ± 0.633*
Increase in vascularity	2.500 ± 0.837	1.833 ± 0.753	1.333 ± 0.516*
Decreased collagen stainability	2.667 ± 0.516	1.500 ± 0.548*	1.500 ± 0.837*

Note: Values are Mean ± SD; *: $p < 0.05$ *versus* MSN group, **: $P < 0.01$ *versus* MSN group, ***: $p < 0.001$ *versus* MSN group.

Supplementary Table 3. List of reagents or resources used in the study.

REAGENT OR RESOURCE	SOURCE	IDENTIFIER
Lymphoprep	STEMCELL Technologies	07811
Recombinant Rat M-CSF	Peprotech	400-28
Lipopolysaccharides	Sigma-Aldrich	L2880
Recombinant mouse GDF-5	R&D system	853-G5
RPMI 1640 medium	Solarbio	31800
Prishin A	Tsbiochem	62499-28-9
Dimethyl sulfoxide	Solarbio	D8371
Phorbol 12-myristate 13-acetate	Sigma-Aldrich	16561-29-8
Phosphate buffered solution	Solarbio	P1020
Live/dead staining kit	Solarbio	CA1630
Sodium pyruvate	Hyclone	SH30239.01
Insulin-Transferrin-Selenium	Thermo Fisher Scientific	41400045
L-ascorbic acid 2-phosphate	Sigma-Aldrich	A5960
TGF-β	Peprotech	
Alcian Blue Stain Kit	Solarbio	G1563
Picro Sirius Red Stain Kit	Abcam	AB150681
Masson's Trichrome Stain Kit	Solarbio	G1340
Alizarin Red S solution	Solarbio	G1450
Safranine O-Fast Green FCF Cartilage Stain Kit	Solarbio	G1371
Mounting Medium with DAPI	ZSGB-BIO	ZLI-9557
Dexamethasone	Sigma-Aldrich	D8893
Dialysis bag	Solarbio	YA1077
DMEM	Hyclone	SH30021.01B
Fetal bovine serum (FBS)	Thermo Fisher Scientific	10099-141
SYBR Green Supermix	Thermo Fisher Scientific	4385612
Prime Script RT Reagent Kit	Takara	RR037B
Trizol Reagent	Thermo Fisher Scientific	15596026
Trypsin-EDTA	Hyclone	SH30042.01

b-Glycerophosphate	APEXBIO	13408-09-8
L-Ascorbic acid	Sigma-Aldrich	A5960
Penicillin/streptomycin	Thermo Fisher Scientific	15070063
Collagenase Type I	Thermo Fisher Scientific	17100017
Dispase	Roche	10269638001
RIPA Buffer	Thermo Fisher Scientific	89900
Protease/Phosphatase Inhibitor Cocktail	Thermo Fisher Scientific	87786
Pierce BCA protein assay kit	Thermo Fisher Scientific	23225
L-Glutamine (200mM)	Thermo Fisher Scientific	25030081
DAB peroxidase substrate kit	ZSGB-BIO	ZLI-9017

Supplementary Table 4. List of rat primers used in the study.

TARGET	FOR QPCR DETECTION	
Cd206	FORWARD	GACAGACGGACGAGGAGTTCATTATAC
	REVERSE	CCACCAATCACAACAACACAGTCAAC
Cd163	FORWARD	TTAGAATCACAGCATGGCACAGGTC
	REVERSE	CCACAAGAGGAAGGCAATGAGAAGG
Arg1	FORWARD	AGAGGAGGTGACTCGTACTGTGAAC
	REVERSE	TCTGGCTTATGATTACCTTCCCGTTTC
Il6	FORWARD	AGTTGCCTTCTTGGGACTGATGTTG
	REVERSE	GGTATCCTCTGTGAAGTCTCCTCTCC
Inos	FORWARD	TCTTGGAGCGAGTTGTGGATTGTTC
	REVERSE	AGTGATGTCCAGGAAGTAGGTGAGG
Il1β	FORWARD	AATCTCACAGCAGCATCTCGACAAG
	REVERSE	TCCACGGGCAAGACATAGGTAGC
Il10	FORWARD	GGCAGTGGAGCAGGTGAAGAATG
	REVERSE	TGTCACGTAGGCTTCTATGCAGTTG
Stat1	FORWARD	TCGCACCTTCGTCCTCTTCCAG
	REVERSE	TTCACCAACAGTCTCAGCTTCACAG
β-actin	FORWARD	ATCGTGGGCCGCCCTAGGCA
	REVERSE	TGGCCTTAGGGTTCAGAGGGG

Supplementary Table 5. List of human primers used in the study.

TARGET	FOR QPCR DETECTION	
ARG-1	FORWARD	TGGACAGACTAGGAATTGGCA
	REVERSE	CCAGTCCGTCAACATCAAAACT
IL-6	FORWARD	TACCCCCAGGAGAAGATTCCA
	REVERSE	CCGTCGAGGATGTACCGAATT
IL-1β	FORWARD	ACCTATCTTCTTCGACACATGGG
	REVERSE	GAGGTGGAGAGCTTTCAGTTCAT
TNF-α	FORWARD	CTGGTATGGACCCATCTATCTGG
	REVERSE	CAGGGCAAATGATCCCAAAGTAGA
GAPDH	FORWARD	ATGGGGAAGGTGAAGGTCG
	REVERSE	GGGGTCATTGATGGCAACAATA

Supplementary Table 6. List of primary and secondary antibodies used in the study.

REAGENT OR RESOURCE	SOURCE	IDENTIFIER	USES
**Antibodies**			
Rabbit-polyclonal anti-iNOS	Proteintech	18985-1-AP	WB (1:1000), IF of tissue (1:100), IF of cell (1:300)
Rabbit-polyclonal anti-CD206	Proteintech	18704-1-AP	WB (1:1000), IF of tissue (1:100), IF of cell (1:300)
Mouse-monoclonal anti-IL-6	Abcam	AB9324	WB (1:1000), IHC (1:200)
Rabbit-monoclonal anti-Arg-1	Cell Signaling Technology	#93668	WB (1:1000)， IF of tissue (1:100)
Mouse-monoclonal anti-CD68	Bio-Red	MCA341B	IF of tissue (1:100), IF of cell (1:300)
Rabbit-monoclonal anti-JAK1	Cell Signaling Technology	#50996	WB (1:1000)
Rabbit-monoclonal anti-Phospho-STAT1	Cell Signaling Technology	#8826	WB (1:1000), IF of tissue (1:100), IF of cell (1:300)
Rabbit- monoclonal anti-STAT1	Cell Signaling Technology	#14994	WB (1:1000)
Rabbit- polyclonal anti-Col II	Proteintech	28459-1-AP	IF of tissue (1:100)
Rabbit- polyclonal anti-Aggrecan	Proteintech	13880-1-AP	IF of tissue (1:100)
Rabbit-monoclonal anti-Tenascin-C	Abcam	AB108930	IF of tissue (1:100)
Rabbit-polyclonal anti-Fibromodulin	Proteintech	13281-1-AP	IF of tissue (1:100)
Rabbit- polyclonal anti-Osteocalcin	Proteintech	23418-1-AP	WB (1:1000)
Rabbit-Polyclonal anti-Osterix	Invitrogen	PA5-40509	WB (1:1000)
Rabbit- monoclonal anti-Phospho-mTOR	Cell Signaling Technology	#5536	WB (1:1000), IF of tissue (1:100)
Rabbit-monoclonal anti-mTOR	Cell Signaling Technology	#2983	WB (1:1000)
Rabbit-polyclonal anti-Tenomodulin	Abcam	AB203676	IF (1:100)
Mouse-monoclonal anti-TNF-α	Abcam	AB1793	IHC (1:100)
Mouse anti-ACTIN	ZSGB-BIO	TA-09	WB (1:3000)
Rabbit-polyclonal anti-Beta TUBULIN	Proteintech	10068-1-AP	WB (1:3000)
Mouse-monoclonal anti-VINCULIN	Proteintech	66305-1-lg	WB (1:3000)
Rabbit-monoclonal anti-GAPDH	Cell Signaling Technology	#5174	WB (1:3000)
HRP-linked anti-mouse IgG	ZSGB-BIO	ZB-2301	WB (1:5000)
HRP-linked anti-rabbit IgG	ZSGB-BIO	ZB-2305	WB (1:5000)
FITC-labeled goat anti-mouse IgG (H + L)	ZSGB-BIO	ZF-0312	IF (1:300)
FITC-labeled goat anti-rabbit IgG (H + L)	ZSGB-BIO	ZF-0311	IF (1:300)
Rhodamine labeled goat anti-mouse IgG (H + L)	ZSGB-BIO	ZF-0313	IF (1:300)
Rhodamine labeled goat anti-rabbit IgG (H + L)	ZSGB-BIO	ZF-0316	IF (1:300)
Horseradish enzyme labelled goat anti-rabbit IgG	ZSGB-BIO	PV9001	IHC
Horseradish enzyme labelled goat anti-mouse IgG	ZSGB-BIO	PV9002	IHC

IF: Immunofluorescence; WB: Western blotting; IHC: Immunohistochemistry

Supplementary Table 7. Software.

Software	
ChemDraw 20.0	https://www.chemdraw.com.
Image J (v1.53k)	https://imagej.nih.gov
Graph Pad Prism 9.00	https://www.graphpad.com
Micro-CT Evaluation CTAnsoftware (version 1.15)	http://www.blue-scientific.com/bruker-micro-ct-software
NRecon	http://www.blue-scientific.com/bruker-micro-ct-software
CTvox	http://www.blue-scientific.com/bruker-micro-ct-software
OMNI Specta	https://www.thermofisher.cn
LAS X 3.0	https://www.leica-microsystems.com.cn
Origin2021	https://www.originlab.com/
NIS-Elements	https://www.nis-elements.com
ZEN 2.3	https://www.zeiss.com.cn
